# Elucidating the coordination of mitochondrial maintenance, functional preservation and glycolytic metabolism in H_2_O_2_-induced beef color stability: an integrated proteomic and biochemical investigation

**DOI:** 10.1016/j.fochx.2026.104537

**Published:** 2026-09-28

**Authors:** Cai-Yun Li, Xiao-Qian Duan, Xin Yu, Yu-Bin Zhang

**Affiliations:** College of Food Science and Engineering, Gansu Agricultural University, Lanzhou 730070, China

**Keywords:** Mitochondrial metabolism, Proteomics, Oxidative stress, Glycolysis, HIF-1 signaling pathway, Postmortem beef

## Abstract

This study investigated the dose-dependent effects of exogenous H_2_O_2_ (300, 500, and 1000 μmol/L) on color stability, mitochondrial function, and proteomic profiles in postmortem beef. Among the concentrations tested, 500 μmol/L H_2_O_2_ most effectively enhanced color stability, as reflected by increased redness and oxymyoglobin proportion and decreased metmyoglobin formation. This treatment also promoted antioxidant enzyme activities, attenuated pH decline, and maintained mitochondrial electron transport chain complex activities. Quantitative proteomic analysis revealed that these beneficial effects were orchestrated by coordinated upregulation of metabolic pathway and HIF-1 signaling proteins, with mitochondrial translation factor GFM1 and lactate dehydrogenase B (LDHB) emerging as central hubs in a protein-protein interaction network. These findings suggest a previously unrecognized adaptive mechanism coupling mitochondrial maintenance, functional preservation, and glycolytic flux regulation under oxidative stress.

## Highlights

• 500 μmol/L H_2_O_2_ significantly enhances beef color stability during postmortem aging.
• Moderate H_2_O_2_ improves antioxidant capacity and maintains mitochondrial ETC activities.
• GFM1 and LDHB coordinate mitochondrial maintenance and glycolysis for color stability.

## 1 Introduction

Beef color is a critical determinant of consumer acceptance and market value, serving as a primary visual indicator of freshness and quality (Gagaoua et al., 2020). Its stability during postmortem storage is a complex trait influenced by multifaceted biochemical processes, including myoglobin redox chemistry, mitochondrial activity, and the muscle's antioxidant capacity (Prommachart et al., 2020; Tuell et al., 2021). Postmortem muscle undergoes a metabolic shift from aerobic respiration to anaerobic glycolysis, leading to pH decline and generating reactive oxygen species (ROS) that can promote the oxidation of oxymyoglobin (OxyMb) to metmyoglobin (MetMb), the brown pigment responsible for color deterioration (Guo et al., 2025; Wang et al., 2025).

Although the role of oxidative stress in meat quality has been extensively studied, a critical knowledge gap remains regarding its dose-dependent effects on color stability and the underlying molecular mechanisms. Recently, HIF-1α signaling has been established as a core regulator of postmortem energy metabolism and meat color. Gao et al. (2025a) revealed that HIF-1 mediates the dynamic coordination of glycolysis and mitochondrial electron transport chain activity to affect tan sheep meat color, while Gao et al. (2025b) further identified LDHA, PGK1, HSPB6, and HSP90B1 as HIF-1α regulated protein biomarkers governing early energy metabolism using the CARS algorithm. Zhu et al. (2024) demonstrated that AMPK and HIF-1α form a feedback loop to regulate mitochondrial efficiency and energy metabolism in yak meat, with HIF-1α activation suppressing mitochondrial function. Recent evidence further indicates that HIF-1α-mediated ferroptosis negatively impacts meat color and water-holding capacity in postmortem yak muscle (Zhu et al., 2025). Collectively, these studies consistently indicate that hypoxia-driven HIF-1α signaling promotes glycolysis but inhibits mitochondrial activity, which is detrimental to meat color stability. However, it remains unknown whether oxidative stress-induced HIF-1 signaling can exert a distinct regulatory pattern to simultaneously maintain mitochondrial function, balance glycolytic flux, and improve beef color stability. To address this knowledge gap, it is essential to understand how different concentrations of H_2_O_2_ influence postmortem muscle metabolism and color stability.

Based on previous research findings, exogenous H_2_O_2_ exerts dose-dependent divergent biological effects in postmortem skeletal muscle, broadly classified into high (≥ 10  mM), moderate, and low (≤ 300  μmol/L) concentration ranges. High-dose H_2_O_2_ provokes severe oxidative stress, expediting postmortem glycolytic progression, initiating mitochondrial apoptotic cascades, and aggravating myofibrillar fragmentation, these biochemical changes effectively promote meat tenderization yet compromise beef color stability, as validated by multiple prior investigations (Wang et al., 2018; Yan et al., 2022; Yang et al., 2024). In contrast, a substantial body of non-meat biological studies has demonstrated that moderate-level oxidative stress induced by appropriate H_2_O_2_ concentrations can elicit typical hormesis-mediated protective effects against oxidative damage (Calabrese, 2016). However, whether such favorable hormetic regulation operates in postmortem beef to preserve or enhance color stability remains entirely unknown. For low-concentration H_2_O_2_ (≤ 300  μmol/L), existing evidence including a 200 μmol/L treatment trial confirms its capacity to accelerate glycolysis and facilitate tenderization without conferring beneficial impacts on meat color performance (Chen et al., 2022). Nevertheless, it is unclear whether a moderate concentration, specifically around 500  μmol/L, could trigger adaptive responses that improve color stability, or whether such effects would fundamentally differ from the destructive metabolic pathways induced by high-dose H_2_O_2_ (≥  10 mM). Therefore, we hypothesized that moderate oxidative stress may induce unique adaptive remodeling at metabolic and proteomic levels to enhance beef color stability. The present study was designed to test this hypothesis.

Central to this oxidative balance are mitochondria, which are not only the primary source of ROS but also key organelles whose postmortem integrity is intimately linked to color stability (Ramos et al., 2024; Yan et al., 2022; Zhu et al., 2023). The rate and extent of mitochondrial dysfunction after death significantly influence the metabolic cascade, including substrate depletion, proton accumulation, and ultimately, the rate of pH decline (Krauskopf et al., 2025; Krauskopf et al., 2026). More importantly, the activities of mitochondrial electron transport chain (ETC) complexes can directly and indirectly affect the redox state of myoglobin (Kiyimba et al., 2022). Sustained ETC activity may help maintain a reducing environment, delaying OxyMb oxidation, while its collapse can exacerbate oxidative stress (Bu et al., 2024; Wang et al., 2025). Mitochondrial function critically depends on the integrity of its own protein synthesis machinery, as mtDNA encodes core subunits of the ETC that are translated by mitochondrial ribosomes under the regulation of translation factors such as elongation factor GFM1 (Ahmad et al., 2025; Rackham et al., 2025). The interplay between externally induced oxidative stress and the inherent metabolic function of mitochondria likely forms a decisive axis regulating the color trajectory of beef. Although proteomic analyses have begun to uncover associations between mitochondrial protein expression and color traits (Gagaoua et al., 2020; Krauskopf et al., 2025), the specific molecular mechanisms through which moderate oxidative stress modulates the mitochondrial proteome, including proteins involved in translation, metabolic pathways, and stress signaling, as well as the functional consequences for electron transport chain activity and color stability, remain to be fully elucidated.

However, it remains unknown whether oxidative stress-induced HIF-1 signaling can exert a distinct regulatory pattern that simultaneously preserves mitochondrial integrity, balances glycolytic flux, and enhances beef color stability—an effect that has not been systematically characterized across pro-oxidative, hormetic, and cytotoxic dose ranges. We hypothesized that a moderate H_2_O_2_ concentration (500 μmol/L) would induce a unique adaptive remodeling at both metabolic and proteomic levels, potentially involving a coordinated network centered on the mitochondrial translation factor GFM1 and lactate dehydrogenase B (LDHB). To test this hypothesis, we systematically investigated the dose-dependent effects of H_2_O_2_ (300, 500, and 1000 μmol/L) on color stability, pH, antioxidant capacity, glycolytic enzyme activities, and mitochondrial ETC complex function during 48 h of postmortem aging. Furthermore, quantitative proteomic analysis was employed on beef treated with the optimal concentration to identify differentially abundant proteins and associated pathways, aiming to elucidate how moderate oxidative stress orchestrates mitochondrial maintenance, functional preservation, and metabolic adaptation to improve fresh beef color stability.

## 2 Materials and methods

### 2.1 Sample preparation

A total of thirty-two (*n* = 32) 1.5-year-old Simmental yellow cattle bulls (with an average live weight of 550 ± 30 kg) were sourced from a commercial farm and slaughtered humanely under standard commercial conditions at the Qilian Muge Industrial Co. Ltd. slaughterhouse (Zhangye, Gansu, China) by trained slaughterhouse staff. All procedures involving animals were approved by the Institutional Animal Care and Use Committee of Gansu Agricultural University (Project No. 2023-2-159).

The longissimus dorsi muscle was excised from the 12th thoracic to 5th lumbar vertebra at 1 h postmortem. The muscle from each animal was then divided, and external fat and connective tissue were meticulously removed. Each muscle was subsequently cut into portions (approximately 100 ± 5 g each, with a thickness of approximately 3–4 cm to allow direct pH probe insertion) for treatment. The portions were randomly assigned to one of four treatment groups (*n* = 8 biological replicates per group) to ensure a statistically robust design: the control group was injected with 0.9% sterile physiological saline, while the three treatment groups were injected with 300 μmol/L, 500 μmol/L, and 1000 μmol/L H_2_O_2_ solutions, respectively. All solutions were injected uniformly into the beef samples at a meat-to-solution ratio of 20:1 (*w*/*v*) using a multi-needle injector, followed by careful massage for approximately 30 s to ensure uniform distribution of the injected solution without exudation. The injected samples were immediately transported to the laboratory under controlled low-temperature conditions using dry ice to maintain chilling (0–4 °C) without freezing. All samples remained in a chilled, unfrozen state, no freeze-thaw cycle occurred before incubation at 4 °C, thereby avoiding artifacts from mitochondrial damage or protein denaturation. Upon arrival, samples were placed in vacuum-packaging bags (without vacuum sealing) and stored at 4 °C for 6, 12, 24, and 48 h to allow myoglobin oxygenation (blooming) on the meat surface under aerobic conditions. At each designated time point, samples were retrieved for the analysis of fundamental quality indicators. Subsequently, the samples were frozen in liquid nitrogen and stored at −80 °C for further biochemical analysis.

### 2.2 pH

The pH assay was referred to Li et al. (2025a) with some modifications. The pH of beef samples was measured by directly inserting a handheld pH meter probe (205, Testo Company, Germany) into each steak (approximately 100 ± 5 g) to a depth of approximately 2–3 cm, and the reading was recorded after stabilization. Six measurements were taken from different locations of each steak and averaged.

### 2.3 Color index

The color values were recorded each postmortem time on beef samples that were referred to Guo et al. (2025) with some modifications. Color measurements were collected using a CR-410 colorimeter (Illuminate A, 10° observer, 8 mm diameter aperture, Konica Minolta Sensor Corporation, Osaka, Japan), which was standardized with white tile before each use. A total of six readings were taken on each sample and averaged. CIE L* values (lightness), a* values (redness), and b* values (yellowness) data were collected.

### 2.4 Proportions of myoglobin redox forms

An aliquot of 5 g of minced muscle was devoid of external fat and connective tissue, homogenized with 25 mL of cold 0.04 M phosphate buffer (pH 6.8) for 30 s using a superfine homogenizer (Ultra Turrax T18 Basic, IKA, Germany). The homogenate was then centrifuged at a speed of 10,000 ×g at 4 °C for 30 min. The supernatant was filtered with Whatman No.1 filter paper. The absorbance was measured at 525, 545, 565 and 572 nm on a spectrophotometer (TU-1810, PERSEE, Beijing, China). The relative contents of deoxymyoglobin (DeoMb), oxymyoglobin (OxyMb) and metmyoglobin (MetMb) were determined according to the method proposed by Krzywicki (1982).

DeoMb (%) = (0.369 R_1_ + 1.140 R_2_–0.941 R_3_ + 0.015) × 100

OxyMb (%) = (0.882 R_1_–1.267R_2_ + 0.809R_3_–0.361) × 100

MetMb (%) = (−2.541 R_1_ + 0.777R_2_ + 0.800 R_3_ + 1.098) × 100

where R_1_, R_2_, R_3_ are the absorbance ratio of A_572nm_/A_525nm_, A_565nm_/A_525nm_ and A_545nm_/A_525nm_, respectively.

### 2.5 Antioxidant enzyme activity assays

Superoxide dismutase (SOD), glutathione peroxidase (GSH-Px) and catalase (CAT) activities were determined in control and different concentrations of H_2_O_2_ groups, respectively. A 5-g muscle sample was homogenized in 25 mL of 0.05 M phosphate buffer (pH 7.0) for 20 min in an ice bath and then centrifuged at 7000 ×*g* for 20 min at 4 °C using a refrigerated centrifuge (Model 5430 R, Eppendorf, Hamburg, Germany). The enzyme extract solution after filtration of the supernatant with glass wool was used to determine the activity of antioxidant enzymes.

The SOD activity was determined as proposed by Marklund and Marklund (1974) by measuring the inhibition of pyrogallol autoxidation. One unit of enzyme activity is defined as the amount of extract required to inhibit the oxidation rate by 50% compared to the control (no SOD). The CAT activity assay was conducted using a modified version of (Mahecha et al., 2011) method. Catalase activity was measured as the loss of H_2_O_2_ by recording the decrease in absorbance at 240 nm within the first 1 min. One unit of enzyme activity was defined as 1 μmol of H_2_O_2_ degradation catalysed per minute per g of beef tissue at room temperature. The activity of GSH-Px in beef samples was determined using the spectrophotometric method by measuring the oxidation of NADPH according to at 340 nm every 30 s for 3 min at 37 °C, according to Jin et al. (2013). The protein concentration of the samples was determined using a BCA kit (Beyotime, China, Cat: P0012S), and GPx values were expressed in nmol/min/mg protein.

### 2.6 Carbonyl content

The protein carbonyl assay was referred to Sun et al. (2011) with some modifications. Two aliquots of 1 mL of 5 mg/mL sarcoplasmic protein were mixed with 250 μL of cold 50% (*w*/w) trichloroacetic acid (TCA). The precipitates were reacted with 3.2 mM 2,4-dinitrophenylhydrazine (DNPH, D199303, Aladdin, Shanghai, China) dissolved in 3.2 M HCl and 3.2 M HCl as a blank for 30 min at ambient temperature. The precipitates were collected after centrifugation at 12,000 ×*g* (using a refrigerated centrifuge, Model 5430 R, Eppendorf, Hamburg, Germany) for 15 min at 4 °C and then washed 3 times with 3 mL of cold acetone. Both samples were incubated under agitation for 1 h at room temperature and then centrifuged at 4000 ×*g* for 10 min at 4 °C. The solutions were clarified by centrifugation. The carbonyl content was measured by detecting the content of incorporated DNPH in protein hydrazones using a Multiskan SkyHigh (Thermo Fisher Scientific, China) based on the absorption of 22,000 M^−1^ cm^−1^ at 370 nm. The results were expressed as nanomoles of DNPH equivalents per milligram of protein.

### 2.7 Free thiol content

The free thiol content was determined according to Ellman (1959) with some modifications. Sarcoplasmic proteins adjusted to a concentration of 3 mg/mL with 15 mM Tris-HCl buffer (T1503, Sigma-Aldrich, St. Louis, MO, USA) were collected in centrifuge tubes. Sarcoplasmic proteins suspension (0.2 mL) was incubated with 2.5 mL of Tris-Gly-8 M Urea and 0.03 mL of 10 mmol/L DTNB (D8130, Sigma-Aldrich, St. Louis, MO, USA) for 1 h (25 °C). The samples were centrifuged at 4000 ×*g* (using a refrigerated centrifuge, Model 5430 R, Eppendorf, Hamburg, Germany) for 15 min (25 °C). The absorbance was measured at 412 nm (A412) using a Multiskan SkyHigh (Thermo Fisher Scientific, China). The content of Free Thiol Content is expressed as nmol/mg protein, and the molar extinction coefficient is 1.36 × 10^4^. The calculation formula is as follows. Free Thiol Content = 10^6^ × A412/(1.36 × 10^4^ × C) where C is the concentration of protein.

### 2.8 Malondialdehyde (MDA) measurement

MDA content was determined according to the test kit (Solarbio BC0025). MDA condenses with Thio barbituric acid (TBA) at high temperature (100 °C, 60 min) to form a brownish-red color of trimethylchoron (3,5,5-trimethyloxazole-2,4-dione), which has a maximum absorption wavelength of 532 nm. The colorimetric analysis was carried out to estimate the MDA content of the sample. The amount of MDA in the sample can be estimated. The analysis was performed in triplicate.

### 2.9 Measurement of lactate dehydrogenase (LDH), pyruvate kinase (PK), and hexokinase (HK) activities

Take 3 g of frozen meat samples, chop them, and homogenize in 27 mL of ice-salt water solution (8.6%). Centrifuge at 1000 ×*g* for 10 min at 4 °C using a refrigerated centrifuge (Model 5430 R, Eppendorf, Hamburg, Germany), collect the supernatant. LDH activity was measured using a commercial kit (Nanjing Jiancheng Bioengineering Institute, China, Cat: A020–1). HK and PK activities were measured using kits from Beijing Solarbio Technology Co., Ltd. (China, Cat: BC0745 for HK, Cat: BC0540 for PK).

### 2.10 Determination of mitochondrial complex activities

The activities of mitochondrial complexes I, II, III, and IV were determined by assay kits. The pretreatment method was briefly described as follows: precisely weigh 0.1 g of muscle tissue, add 1.0 mL of extraction buffer, and homogenize using an ice-bath homogenizer. Centrifuge at 600 ×*g* for 10 min at 4 °C using a refrigerated centrifuge (Model 5430 R, Eppendorf, Hamburg, Germany). Transfer the supernatant to another centrifuge tube and centrifuge at 11,000 ×*g* for 15 min at 4 °C.

Add 400 μL of extraction buffer to the resulting pellet, followed by ultrasonic disruption (200 W power, 5-s sonication with 10-s intervals, repeated 15 times). The enzymatic activities of the complexes were then determined according to the manufacturer's instructions using the mitochondrial respiratory chain complex I (BC0515), II (BC3230), III (BC3240), and IV (BC0945) activity assay kits (Solarbio, Beijing, China).

### 2.11 Analysis and detection of proteomics

To investigate the molecular mechanisms underlying the observed color stability, for each of the two selected treatment groups (control and 500 μmol/L H_2_O_2_) and for each postmortem time point (6, 24, and 48 h), three biological replicates were randomly selected from the eight available replicates of that group and time point for proteomic profiling. This resulted in a total of 18 samples (2 treatments × 3 time points × 3 replicates = 18). The selection was performed randomly to ensure unbiased representation of each experimental group. These samples were used for protein extraction, digestion, and LC-MS/MS analysis as described below.

#### 2.11.1 Protein extraction and quantification

The beef samples were stored at −80 °C before extracting the total proteins. Frozen muscle tissue (100 mg) was homogenized in 500 μL of 8 M urea containing 5 μL PMSF using an ultrasonic homogenizer (JY92-IIN, Scientz, Ningbo, China) on ice for 45 min. The homogenate was centrifuged at 12,000 ×*g* for 20 min at 4 °C using a refrigerated centrifuge (Model 5430 R, Eppendorf, Hamburg, Germany) and the supernatant was collected. Protein concentration was determined using a BCA Protein Assay Kit (Beyotime, China, Cat: P0012S) according to the manufacturer's instructions. Briefly, samples were diluted 10-fold, mixed with working reagent (A: B = 50:1), incubated at 37 °C for 30 min, and absorbance was measured at 562 nm using a microplate reader. The protein quality was assessed by SDS-PAGE on a 12% gel (POWERPAC 200, Bio-Rad, Hercules, CA, USA) with Coomassie Brilliant Blue staining (Beyotime, China).

#### 2.11.2 SDS-PAGE and quality control

A total of 30 μg of protein from each sample was mixed with 5 × loading buffer, denatured at 100 °C for 5 min, and separated on a 12% SDS-PAGE gel at a constant voltage of 120 V for approximately 90 min using a PowerPac 200 electrophoresis system (Bio-Rad, Hercules, CA, USA). Proteins were visualized using Coomassie Brilliant Blue staining to confirm extraction quality and integrity.

#### 2.11.3 Protein digestion

Protein samples (50 μg) were reduced with 10 mM DTT at 56 °C for 1 h, alkylated with 20 mM iodoacetamide in the dark at room temperature for 40 min, and then quenched with 10 mM DTT. SP3 beads (Cytiva, Marlborough, MA, USA, Cat: 45152105050250) were washed three times with ultrapure water and resuspended to 100 μg/μL. Samples were combined with 10 μL bead suspension and 110 μL ethanol, incubated for 15 min at room temperature with gentle shaking, and separated on a magnetic rack. The beads were washed three times with 500 μL of 80% ethanol and air-dried until the bead surface appeared matte without liquid reflection. Digestion was performed in 300 μL of 50 mM NH₄HCO₃ with trypsin (0.25 μg/μL, Enzyme Research, T240716) at 37 °C overnight (14–18 h) with shaking at 1000 rpm. After digestion, the supernatant was collected on a magnetic rack. The peptides were vacuum-dried and desalted using C18 StageTips (Thermo Fisher Scientific, Waltham, MA, USA). The StageTips were conditioned with 100 μL of methanol, followed by 100 μL of 0.1% formic acid in 80% acetonitrile, and finally 100 μL of 0.1% formic acid in water. The peptide samples were loaded by centrifugation at 1500 ×*g* for 2 min. After washing twice with 100 μL of 0.1% formic acid in water, the peptides were eluted with 100 μL of 0.1% formic acid in 60% acetonitrile, followed by 50 μL of 0.1% formic acid in 80% acetonitrile. The eluates were pooled and vacuum-dried for subsequent LC-MS/MS analysis.

#### 2.11.4 LC-MS/MS analysis

Peptides were reconstituted in 0.1% formic acid and analyzed on a Vanquish Neo UHPLC system coupled to an Orbitrap Exploris 480 mass spectrometer (Thermo Fisher Scientific, Waltham, MA, USA). Samples were loaded onto a pre-column (75 μm × 2 cm, NanoViper C18 3 μm, 100 Å, Thermo Fisher Scientific) and separated on an analytical column (150 μm × 170 mm, Reprosil-Pur 120 C18-AQ 1.9 μm) with the following gradient at a flow rate of 400 nL/min: 0–1.8 min, 6% B; 1.8–2 min, 6.5% B; 2–85 min, 6% to 28% B; 85–106 min, 28% to 38% B; 106–110 min, 38% to 90% B; 110–111 min, 90% to 99% B; 111–120 min, 99% B (mobile phase B: 0.1% formic acid in 80% acetonitrile).

MS data were acquired in data-independent acquisition (DIA) mode using variable isolation windows with 1 *m*/*z* overlap and 60 windows covering the mass range. Full MS scans were performed at a resolution of 120,000 with an AGC target of 300% and maximum injection time of 22 ms over a range of 400–1100 *m*/*z*. DIA scans were acquired with a resolution of 30,000, AGC target of 1000%, maximum injection time of 40 ms, and collision energies of 25, 27, and 30 eV.

#### 2.11.5 Data processing and database search

Raw data were processed using DIA-NN software (Biognosys AG, Switzerland) against the *Bos taurus* UniProt database. Search parameters included: fixed modification of carbamidomethyl (C), variable modifications of oxidation (M) and acetyl (N-terminal), trypsin digestion with up to two missed cleavages, and mass tolerances of 20 ppm for both precursor and fragment ions. The false discovery rate (FDR) for both protein and peptide identification was set to 1%. Proteins were considered for further analysis only if they were identified with at least two unique peptides. This stringent filtering ensures high confidence in the protein identifications and quantifications.

To identify differentially abundant proteins (DAPs) between the H_2_O_2_-treated and control groups at each postmortem time point, the normalized protein intensities were compared using a two-tailed Student's *t*-test. Proteins with a *P* value <0.05 and an absolute fold change ≥1.2 (|log₂FC| ≥ 0.26) were considered differentially expressed. Only those proteins that were consistently up- or down-regulated across all three postmortem time points (6, 24, and 48 h) and met the above criteria in each comparison were defined as common DAPs and used for subsequent bioinformatics analyses. The FDR was estimated using the Benjamini–Hochberg procedure and is provided in Table S1 for reference. However, only the *P* value and fold change thresholds were applied for the initial selection of DAPs. The complete list of common DAPs, including their log_2_ fold changes, *P* values, and FDR values, is presented in Table S1. This relatively lenient threshold was chosen because the study is exploratory and aims to capture potentially biologically relevant proteins that may be missed under a stringent FDR correction, particularly given the limited sample size (*n* = 3 per time point) common in postmortem muscle proteomics. The accompanying FDR values in Table S1 are provided for transparency and allow readers to assess the confidence of each DAP.

#### 2.11.6 Bioinformatics analysis

The gene ontology (GO) enrichment and KEGG pathway analysis for the differentially abundant proteins (DAPs) were performed using the DAVID database (version 6.8), available at https://david.abcc.ncifcrf.gov/. This analysis covered the three major GO categories, biological process (BP), cellular component (CC), and molecular function (MF) as well as KEGG pathway terms. For GO enrichment, a hypergeometric test was applied to identify terms significantly over represented among the DAPs compared to the background proteome. To account for multiple testing, the Benjamini–Hochberg procedure was used to control the FDR. Functional terms with an FDR < 0.05 were considered statistically significant.

Protein-protein interaction (PPI) networks of the DAPs were constructed using STRING database version 11.0 (http://string-db.org). To ensure the reliability and biological relevance of the interactions, the network was stringently filtered. Specifically, only interactions with experimental evidence available in the STRING database (evidence channel: “experimentally determined”), which are derived from techniques such as yeast two-hybrid, co-immunoprecipitation, or biochemical assays, were retained. Interactions predicted solely based on other evidence channels, such as gene co-occurrence, text mining, or neighborhood, were excluded from the analysis. Furthermore, only those experimentally validated interactions with a high confidence score > 0.7 were considered. The resulting PPI network was then visualized and analyzed using Cytoscape software (version 3.9.1).

### 2.12 Statistical analysis

For physicochemical indicators (pH, color, enzyme activities, oxidation markers, etc.), all data are presented as mean ± standard deviation (SD) of eight biological replicates (*n* = 8). All physicochemical assays were performed in triplicate as technical replicates. Normality of data distribution was assessed using the Shapiro–Wilk test prior to parametric analysis. The data were subjected to a two-way analysis of variance (ANOVA) using a general linear model to evaluate the main effects of treatment (Control, 300, 500, and 1000 μmol/L H_2_O_2_), postmortem time (6, 12, 24, and 48 h), and their interaction. The significance of the main effects and the interaction effect was determined at the *P* < 0.05 level. When a significant interaction was found, differences among means were analyzed by Tukey's honest significant difference (HSD) post-hoc test. Significance is denoted in tables and figures using lowercase superscript letters (a − d) to indicate significant differences between treatments at the same aging time, and uppercase superscript letters (A − D) to indicate significant differences over time within the same treatment. Different letters represent statistically significant differences (*P* < 0.05). All statistical analyses for physicochemical data were performed using SPSS Statistics software (version 26; IBM Corp., Armonk, NY, USA).

For proteomic data analysis, the identification of differentially abundant proteins (DAPs) and the definition of common DAPs across time points were performed using the criteria described in Section 2.11.5 (*P* value <0.05, absolute fold change ≥1.2, and consistent regulation across 6, 24, and 48 h). The Benjamini–Hochberg false discovery rate (FDR) estimates for each DAP are provided in Table S1. All statistical analyses for proteomic data were performed using R software (version 4.3.1).

Pearson correlation analysis was conducted to examine the relationships between the abundance of 13 key differentially abundant proteins (DAPs) and all measured physicochemical indicators. The protein abundance data used in this analysis were obtained from the proteomic profiling of samples from the 500 μmol/L H_2_O_2_ treatment and control groups at 6, 24, and 48 h postmortem, as detailed in Section 2.11. The corresponding physicochemical indicator data were derived from the same individual samples subjected to proteomic analysis, ensuring paired comparisons. Correlation coefficients (r) and corresponding *P* values were calculated using the cor function in R software (version 4.3.1). The resulting correlation matrix was visualized as a heatmap using the ggplot2 package, with hierarchical clustering applied to both rows and columns to group variables with similar correlation patterns. For proteomic data, DAPs were identified as described in Section 2.11.5. The use of *P* < 0.05 and |log₂FC| ≥ 0.26 without initial FDR filtering was chosen for exploratory purposes to balance sensitivity and specificity. FDR values are provided in Table S1 for reference.

A comprehensive summary of method validation parameters (linearity, LOD, LOQ, and precision as RSD) for all quantitative analytical methods is provided in Table S3 in the Supplementary Materials.

## 3 Results

### 3.1 Effects of H_2_O_2_ on pH and color stability of postmortem beef

The pH values of beef samples during postmortem aging are shown in Fig. 1. In the control and 300 μmol/L H_2_O_2_ groups, pH decreased significantly from 6 h to 24 h (*P* < 0.05) and then remained stable between 24 h and 48 h (*P* > 0.05). In contrast, in the 500 and 1000 μmol/L H_2_O_2_ groups, pH showed a gradual but significant decline throughout the 48 h storage period (*P* < 0.05). Compared with the control group, the 300 μmol/L treatment resulted in lower pH values at most time points (*P* < 0.05), indicating an accelerated acidification. Conversely, the 500 and 1000 μmol/L treatments led to significantly higher pH values than the control at all time points (*P* < 0.05), with the 1000 μmol/L group exhibiting the highest pH values, followed by the 500 μmol/L group. Thus, only the 500 μmol/L and 1000 μmol/L concentrations of H_2_O_2_ attenuated the postmortem pH decline, whereas the 300 μmol/L exacerbated acidification.

**Fig. 1:**
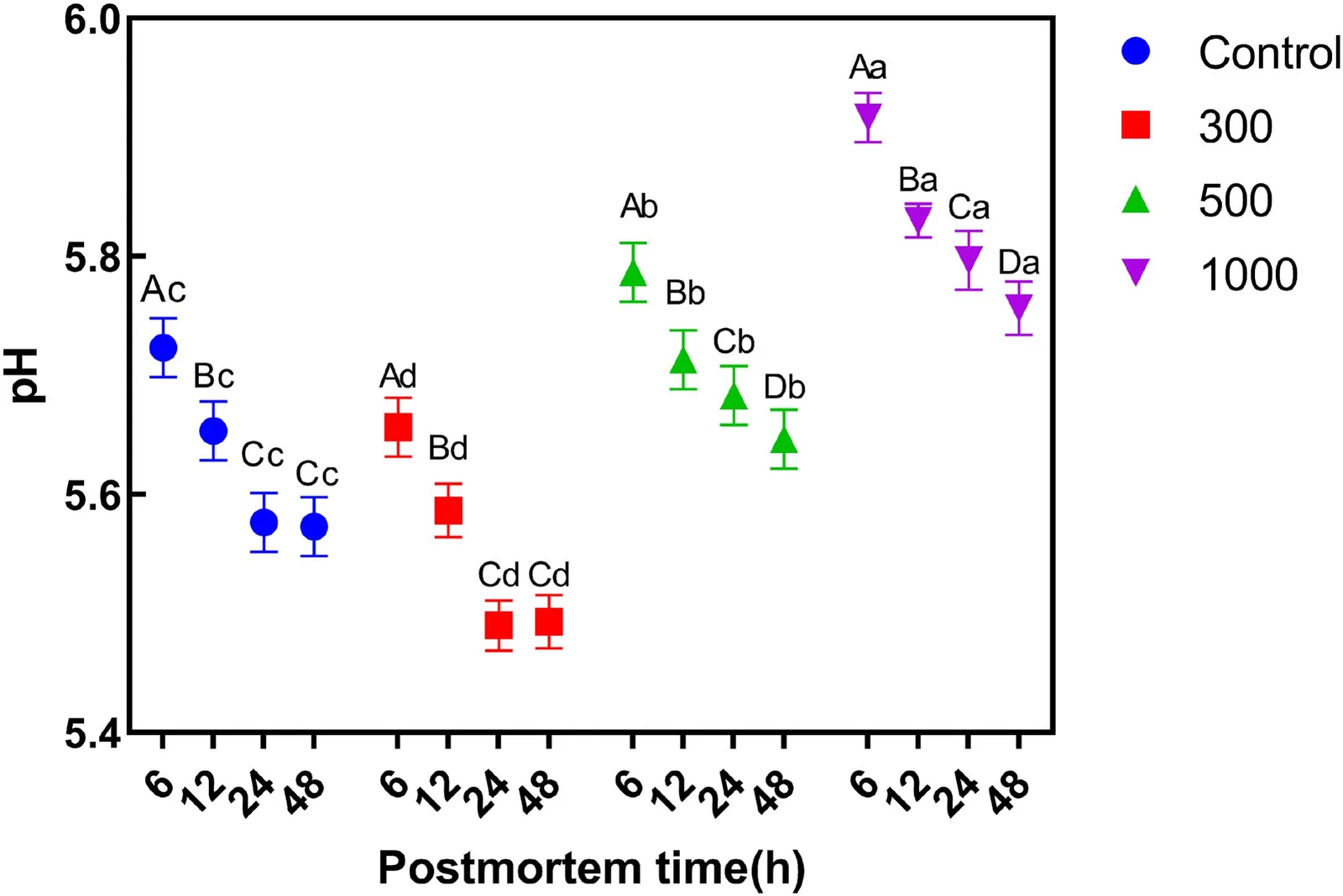
Changes in pH values of beef samples injected with 300, 500, and 1000 μmol/L H_2_O_2_ during 48 h of postmortem aging. Values are mean ± SD (*n* = 8). Lowercase letters (a-d) indicate significant differences between treatments at the same time; uppercase letters (A-D) indicate significant differences over time within a treatment (*P* < 0.05).

The effects on color and myoglobin forms are shown in Table 1. The L* value was significantly higher in the 500 μmol/L H_2_O_2_ group from 12 h to 48 h compared to other groups (*P* < 0.05). The a* value was consistently highest in the 500 μmol/L group at each time point (*P* < 0.05), the b* value was highest in the 1000 μmol/L group (*P* < 0.05). Furthermore, the proportion of OxyMb was significantly highest in the 500 μmol/L group throughout storage (*P* < 0.05), reaching 29.48% at 48 h, which was more than double that of the control group (13.87%). Conversely, the formation of MetMb was significantly lowest in the 500 μmol/L group at 24 h and 48 h (*P* < 0.05). The DeoMb proportion was lowest in the 500 μmol/L group (*P* < 0.05). Notably, these findings affirm that 500 μmol/L H_2_O_2_ was the most effective concentration for improving color stability, as it promoted OxyMb retention, minimized MetMb formation, and resulted in the highest lightness and redness.

**Table 1:** Color parameters and myoglobin redox forms in beef injected with H_2_O_2_ during postmortem storage.

Parameter	H_2_O_2_ Concentration/μmol/L	Storage time/h			
		6	12	24	48
L*	Control	40.05 ± 0.43^Aa^	37.88 ± 0.53^Bc^	36.90 ± 0.35^Cc^	35.67 ± 0.62^Dc^
	300	33.74 ± 0.42^Bd^	37.18 ± 0.45^Ad^	30.69 ± 0.50^Cd^	29.95 ± 0.34^Dd^
	500	39.06 ± 0.37^Cb^	44.89 ± 0.48^Aa^	43.25 ± 0.44^Ba^	43.24 ± 0.38^Ba^
	1000	36.75 ± 0.51^Cc^	39.47 ± 0.58^Ab^	38.48 ± 0.48^Bb^	38.12 ± 0.32^Bb^
					
a*	Control	21.45 ± 0.36^Ac^	20.37 ± 0.28^Bc^	20.07 ± 0.37^Bc^	18.9 ± 0.21^Cc^
	300	20.67 ± 0.31^Ad^	18.68 ± 0.23^Bd^	18.17 ± 0.21^Bd^	17.38 ± 0.20^Cd^
	500	27.57 ± 0.32^Aa^	26.55 ± 0.35^Ba^	24.29 ± 0.34^Ca^	21.30 ± 0.24^Da^
	1000	23.81 ± 0.26^Ab^	22.61 ± 0.27^Bb^	21.06 ± 0.22^Cb^	20.11 ± 0.22^Db^
					
b*	Control	6.25 ± 0.45^Cd^	6.41 ± 0.29^Bd^	6.73 ± 0.32^Bd^	7.68 ± 0.34^Ad^
	300	7.25 ± 0.26^Cc^	7.59 ± 0.33^Bc^	7.83 ± 0.20^Bc^	8.08 ± 0.34^Ac^
	500	8.05 ± 0.34^Cb^	8.42 ± 0.34^Bb^	8.74 ± 0.33^Ab^	8.79 ± 0.33^Ab^
	1000	9.29 ± 0.35^Ca^	10.05 ± 0.32^Ba^	10.09 ± 0.29^Ba^	10.62 ± 0.33^Aa^
					
OxyMb	Control	19.15 ± 1.04^Ad^	17.72 ± 0.72^Bc^	15.77 ± 0.52^Cc^	13.87 ± 0.72^Dc^
	300	23.36 ± 0.37^Ac^	22.09 ± 0.47^Bb^	21.26 ± 0.60^Cb^	19.95 ± 0.20^Db^
	500	34.93 ± 0.65^Aa^	32.71 ± 1.45^Ba^	30.39 ± 0.96^Ca^	29.48 ± 0.62^Da^
	1000	25.35 ± 0.42^Ab^	24.19 ± 0.51^Bb^	22.39 ± 0.70^Cb^	20.95 ± 0.89^Db^
					
MetMb	Control	34.38 ± 0.97^Db^	36.14 ± 1.17^Cb^	40.96 ± 1.29^Bb^	41.63 ± 0.80^Ab^
	300	33.26 ± 1.40^Dc^	35.00 ± 1.06^Cb^	41.49 ± 1.04^Ba^	44.88 ± 1.57^Aa^
	500	32.53 ± 0.52^Dd^	34.92 ± 1.19^Cc^	37.30 ± 1.31^Bc^	39.57 ± 0.58^Ac^
	1000	35.31 ± 0.56^Da^	38.94 ± 0.74^Ca^	40.52 ± 0.49^Bb^	43.90 ± 2.14^Aa^
					
DeoMb	Control	46.47 ± 1.89^Aa^	46.14 ± 1.31^Aa^	43.26 ± 0.85^Ca^	44.49 ± 1.42^Ba^
	300	43.37 ± 1.77^Ab^	42.89 ± 0.86^Ab^	37.24 ± 0.89^Bb^	35.17 ± 1.37^Cb^
	500	32.54 ± 0.92^Ad^	32.38 ± 0.45^Ad^	32.31 ± 1.57^ABd^	30.93 ± 0.86^Cc^
	1000	39.33 ± 0.95^Ac^	36.86 ± 1.23^Bc^	37.09 ± 0.81^Cc^	35.14 ± 1.51^Db^

Values are mean ± SD (*n* = 8). Lowercase letters (a-d) indicate significant differences between treatments at the same time; uppercase letters (A-D) indicate significant differences over time within a treatment (*P* < 0.05). OxyMb: Oxymyoglobin; MetMb: Metmyoglobin; DeoMb: Deoxymyoglobin.

### 3.2 Response of the endogenous antioxidant system and oxidation markers to H_2_O_2_ treatment

The activities of antioxidant enzymes (GPX, SOD, CAT) and the levels of oxidation markers (protein carbonyl, free thiol, and MDA) in beef muscle following H_2_O_2_ treatment are shown in Fig. 2. The activity of GPX was significantly higher in the 500 μmol/L H_2_O_2_ group than in all other groups at each time point (Fig. 2a, *P* < 0.05). SOD activity declined during storage in all groups, but this decrease was less pronounced in the 500 μmol/L group, which maintained the highest activity from 12 h onwards (Fig. 2b, *P* < 0.05). The response of CAT activity was more variable, with no single treatment consistently showing the highest activity across all time points. However, the 500 μmol/L group showed a strong and stable CAT activity, particularly at the critical mid-storage period (12 h and 24 h), which would contribute to its overall antioxidant capacity (Fig. 2c, *P* < 0.05). Protein carbonyl content increased in all groups over time, with the 300 and 1000 μmol/L treatments showing significant peaks at 24 h that were higher than the control (Fig. 2d, *P* < 0.05). The 500 μmol/L group maintained relatively lower carbonyl levels throughout storage. A progressive decline in free thiol content was observed in all groups during storage (Fig. 2e). The 500 and 1000 μmol/L H_2_O_2_ treatments resulted in significantly lower free thiol content than the control at 24 h and 48 h (*P* < 0.05), indicating protein oxidation. However, at the 500 μmol/L concentration, this negative effect appeared to be outweighed by the concurrent enhancement of antioxidant capacity and metabolic regulation, which led to an overall improvement in color stability. MDA content increased over time in all treatments (Fig. 2f). The 300 μmol/L group consistently exhibited the highest MDA levels at each time point (*P* < 0.05), while the 500 and 1000 μmol/L groups showed lower values than both the control and 300 μmol/L treatments. Thus, results suggested that H_2_O_2_ treatment induced concentration-dependent effects on the antioxidant system and oxidation markers. The 500 μmol/L treatment was associated with higher GPX and SOD activities and lower MDA content, but also with greater loss of free thiols. The 300 μmol/L treatment appeared to promote lipid peroxidation most substantially.

**Fig. 2:**
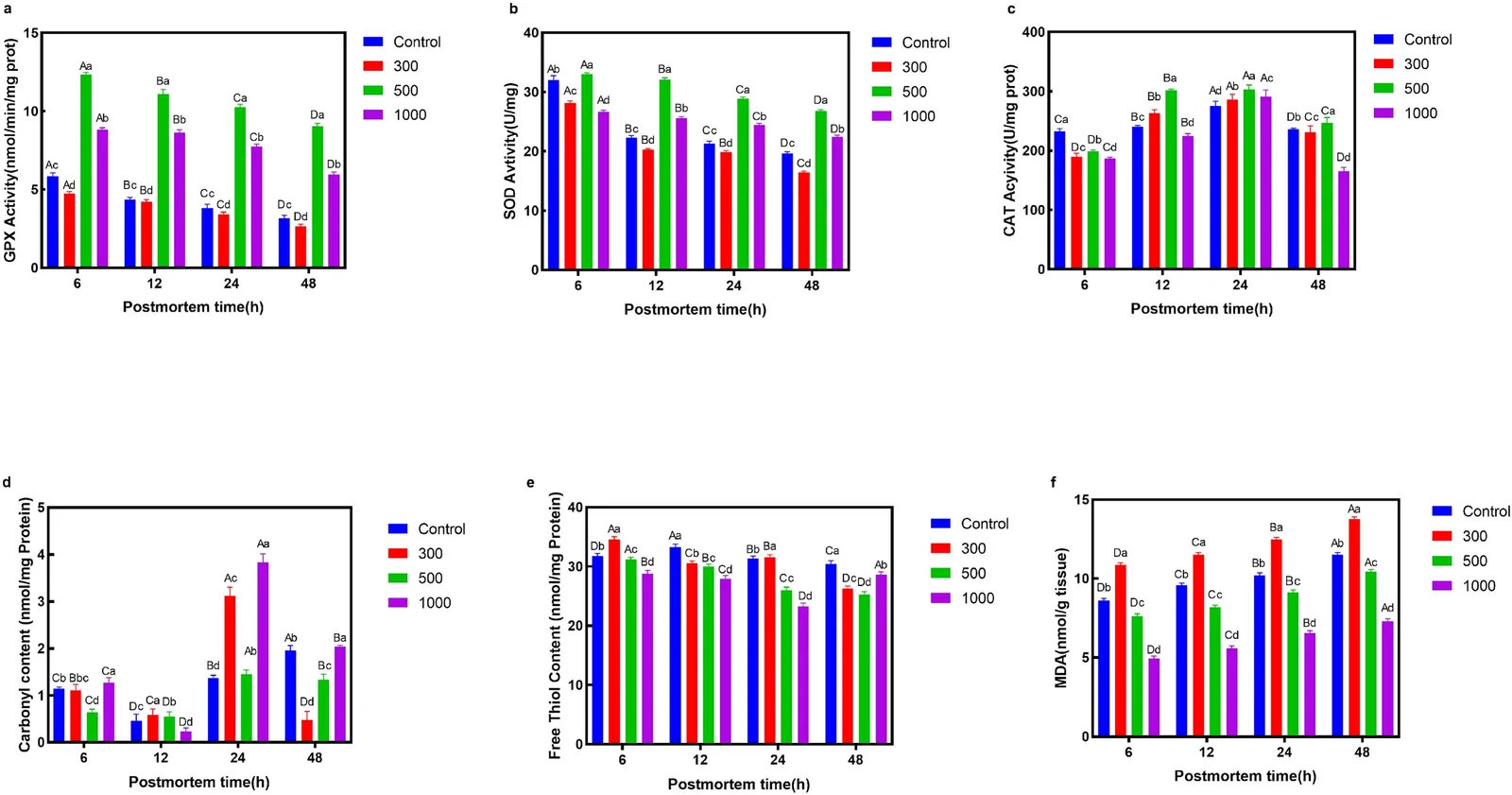
Activities of antioxidant enzymes (GPX, a; SOD, b; CAT, c) and levels of oxidation markers (protein carbonyl, d; free thiol, e; MDA, f) in beef muscle during postmortem storage.Values are mean ± SD (*n* = 8). Uppercase letters (A-D) indicate significant differences over time within a treatment; lowercase letters (a-d) indicate significant differences between treatments at the same time (*P* < 0.05). CAT: catalase; GPX: glutathione peroxidase; SOD: superoxide dismutase; MDA: malondialdehyde.

### 3.3 Activities of key glycolytic enzymes following H_2_O_2_ treatment

The activities of key glycolytic enzymes, namely LDH, PK, and HK, in beef muscle after H_2_O_2_ treatment are presented in Fig. 3. Correspondingly, LDH activity was significantly higher in the 500 μmol/L H_2_O_2_ groups than in the control, 300 and 1000 μmol/L groups throughout the postmortem time (Fig. 3a, *P* < 0.05). In contrast, the control and 300 μmol/L groups exhibited a progressive decline in LDH activity over time (*P* < 0.05). Notably, PK activity was significantly elevated in the 500 μmol/L group, reaching a peak of 144.72 U/g at 24 h, which was higher than the control (75.04 U/g) at the same time point (*P* < 0.05). The 1000 μmol/L group also showed higher PK activity than the control at most time points (Fig. 3b). HK activity was significantly increased in the 500 μmol/L group at 6 h (1517.48 U/g) compared to the control (*P* < 0.05). Although activity decreased at later time points, it remained elevated compared to the other groups (Fig. 3c). These results highlight that, compared to the control group, the 300 μmol/L group, and the 1000 μmol/L beef groups, the 500 μmol/L H_2_O_2_ treatment was associated with enhanced activities of LDH, PK, and HK.

**Fig. 3:**
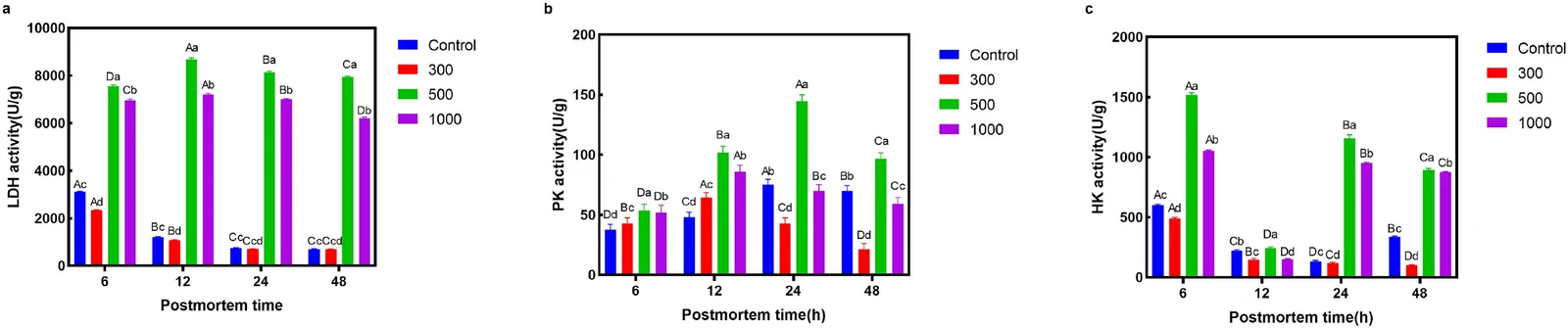
Activities of lactate dehydrogenase (LDH, a), pyruvate kinase (PK, b), and hexokinase (HK, c) in beef muscle injected with H_2_O_2_ during postmortem storage. Values are mean ± SD (*n* = 8). Uppercase letters (A-D) indicate significant differences over time within a treatment; lowercase letters (a-d) indicate significant differences between treatments at the same time (*P* < 0.05).

### 3.4 Modulation of mitochondrial electron transport chain complex activities by H_2_O_2_

The activities of mitochondrial electron transport chain (ETC) complexes (I–IV) in beef muscle under different H_2_O_2_ treatments are shown in Fig. 4. Postmortem time and H_2_O_2_ concentration significantly influenced the activities of all complexes (*P* < 0.05). Complex I activity increased progressively in the 500 μmol/L H_2_O_2_ group, reaching a peak of 121.27 U/mg prot at 48 h, which was higher than all other treatments at that time point (*P* < 0.05). In contrast, the 300 and 1000 μmol/L treatments generally resulted in lower Complex I activity compared to the control (Fig. 4a, *P* < 0.05). Complex II activity was initially inhibited in all H_2_O_2_-treated groups at 6 h and 12 h, but showed recovery by 48 h (Fig. 4b). The 500 μmol/L group achieved the highest final activity (1.73 U/mg prot) at 48 h, which was higher than the control (*P* < 0.05). The activities of Complexes III and IV were higher in the 500 and 1000 μmol/L groups than in the control at 24 h and 48 h (Fig. 4c, d, *P* < 0.05). The 500 μmol/L treatment had the highest activities for both complexes, with peaks of 21.23 U/mg prot (Complex III) and 2.30 U/mg prot (Complex IV) at 24 h. These results showed that the 500 μmol/L H_2_O_2_ treatment was associated with increased activity of Complex I at 12, 24 and 48 h and Complexes II, III, and IV compared to the control at 48 h.

**Fig. 4:**
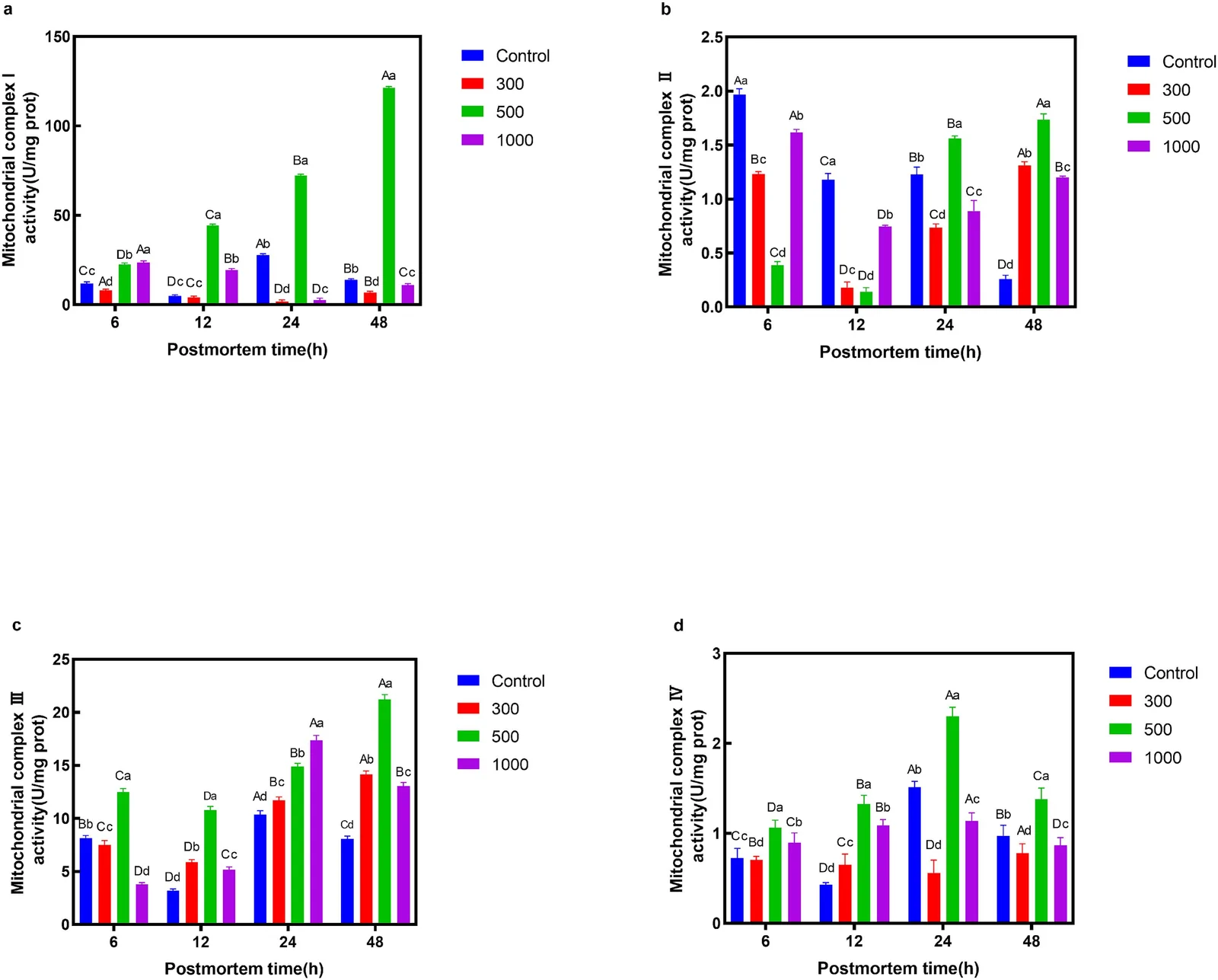
Activities of mitochondrial complexes I (a), II (b), III (c), and IV (d) in beef muscle injected with H_2_O_2_ during postmortem storage. Values are mean ± SD (*n* = 8). Uppercase letters (A-D) indicate significant differences over time within a treatment; lowercase letters (a-d) indicate significant differences between treatments at the same time (*P* < 0.05).

### 3.5 Protein identification and quantification

Based on the above results demonstrating the optimal effects of 500 μmol/L H_2_O_2_ on color stability, antioxidant capacity, and mitochondrial complex activities, samples from the 500 μmol/L H_2_O_2_ treatment group at 6, 24, and 48 h postmortem were selected for quantitative proteomic analysis. The 500 μmol/L concentration was chosen over 300 μmol/L because the latter, despite inducing substantial lipid peroxidation (Fig. 2f), failed to improve color stability (Table 1) and did not elicit the adaptive mitochondrial and antioxidant responses observed at 500 μmol/L (e.g., preserved ETC complex activities, enhanced GPX/SOD). The time points 6, 24, and 48 h were selected because they collectively capture the dynamic proteomic changes underlying the observed color stability within the 48 h postmortem window, during which treatment-dependent differences in color parameters and mitochondrial function became most evident.

As shown in Fig. 5A, a total of 3243 protein groups were identified. Among them, 2507, 2700, 2602, 2715, 2874, and 2833 proteins were detected in the six experimental groups (control and H_2_O_2_-treated, each with three biological replicates at 6, 24, and 48 h postmortem), respectively. Moreover, the Venn diagram in Fig. 5B presented a comparative analysis of the proteomic differences between beef samples treated with 500 μmol/L H_2_O_2_ at different postmortem times. The diagram showed that 2070 proteins (64% of the total) were common to all samples, while 1173 proteins (36% of the total) were unique to specific groups. Among the non-shared proteins, the control group had 12, 13, and 9 unique proteins at 6-, 24-, and 48-h postmortem, respectively. The 500 μmol/L H_2_O_2_ treatment group had 64, 66, and 10 unique proteins at the corresponding time points. These substantial proteomic changes provide a molecular basis for the observed physiological variations in muscle color and antioxidant status. The distinct protein profiles between control and H_2_O_2_-treated groups strongly suggest that the improved color stability is driven by specific alterations in the muscle proteome.

**Fig. 5:**
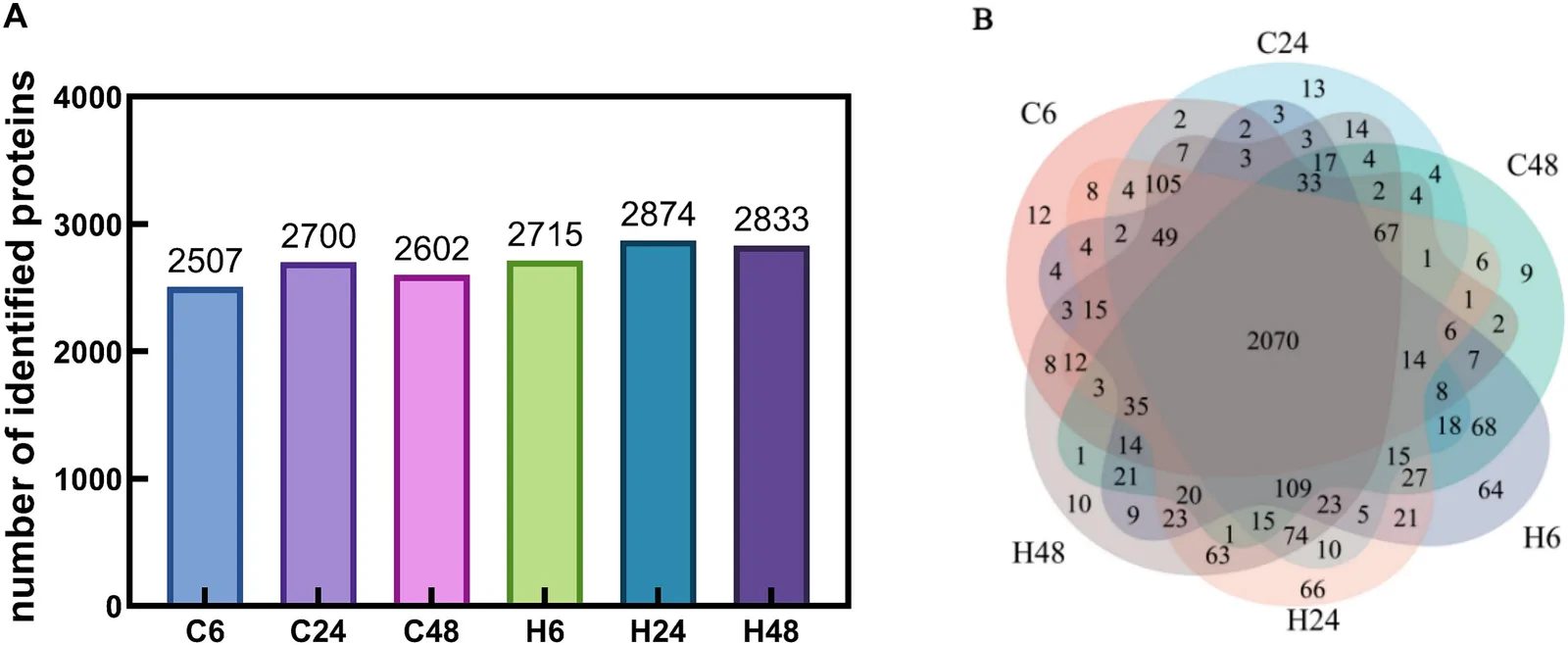
Protein identification and analysis. (A) Number of identified proteins in each sample (*n* = 3 biological replicates per group). Sample labels: C6, C24, C48 represent control groups at 6, 24, and 48 h postmortem; H6, H24, H48 represent H_2_O_2_-treated groups (500 μmol/L) at the corresponding time points. (B) Venn diagram showing the distribution of identified proteins across all six experimental groups. Numbers indicate proteins uniquely or commonly identified among groups.

### 3.6 Analysis of differentially abundant proteins (DAPs)

#### 3.6.1 DAPs and correlations

The principal component analysis (PCA) results revealed significant overall differences in the proteomic profiles among the six beef groups (PC1: 39.7%, PC2: 28.4%). As clearly shown in Fig. 6A, samples within each of the 6 beef groups clustered together, while distinct separation was observed between groups, necessitating further investigation into the composition and categories of DAPs among the six beef samples. As illustrated in Fig. 6B, 99, 97, and 106 DAPs were identified in the comparisons of H6-vs-C6, H24-vs-C24, and H48-vs-C48, respectively. In Fig. 6C, cluster analysis was performed to enhance the visualization of DAP abundance variations across different treatments and time points. Each row was normalized using *Z*-score transformation. Each column represents a sample, and each row represents a protein. Differential abundance levels of the same DAPs in different beef samples are indicated by distinct colors: red for upregulation, blue for downregulation, and white for undetectable expression. Furthermore, vertical clustering in the figure represents sample clustering, while horizontal clustering represents protein clustering. The vertical clustering reveals expression patterns of protein content among samples. These findings highlight substantial alterations in the protein profiles of beef samples treated with 500 μmol/L H_2_O_2_ compared to the control group at 6-, 24-, and 48-h post-mortem. These changes are consistent with the PCA results, confirming significant differences among the various meat sample groups. Therefore, 149 common DAPs were selected as the focus for bioinformatics analysis, aiming to identify potential protein markers associated with beef color quality under 500 μmol/L H_2_O_2_-induced oxidative stress. The key DAPs used for PPI network construction and correlation analysis are summarized in Table 2, and the complete list of all common DAPs is provided in Table S1.

**Fig. 6:**
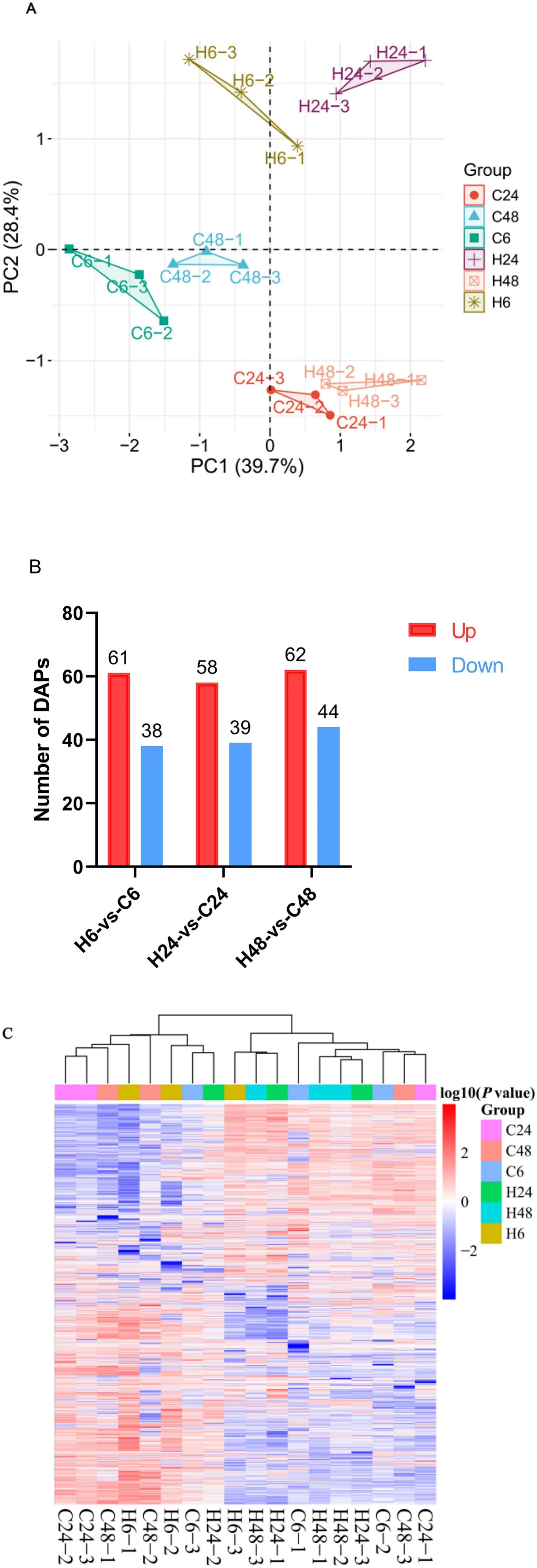
Profiling of differentially abundant proteins (DAPs) between H_2_O_2_-treated (500 μmol/L) and control beef at 6, 24, and 48 h postmortem. Three biological replicates were analyzed per group (*n* = 3). A, Principal component analysis (PCA) showing proteomic profile separation among groups. Sample labels: prefix H/C indicates H_2_O_2_-treated or control; number indicates postmortem time (h); suffix (−1,−2,−3) indicates biological replicates. B, Number of DAPs identified in each comparison (H6 vs. C6, H24 vs. C24, H48 vs. C48). DAPs were defined as proteins with *P* < 0.05 and |log₂FC| ≥ 0.26. C, Heat map of DAP abundance across all samples. Red indicates upregulation; blue indicates downregulation; white indicates undetectable expression. Rows represent individual DAPs; columns represent biological replicates. Hierarchical clustering was applied to both rows and columns. (For interpretation of the references to color in this figure legend, the reader is referred to the web version of this article.)

**Table 2:** Key differentially abundant proteins (DAPs) identified between 500 μmol/L H_2_O_2_-treated and control beef during postmortem aging.

Protein ID	Log_2_FC	*P* value	Protein Description	Gene Name	Protein Function
A0A3Q1LYU1	1.585507042	0.02020944	Protein-serine/threonine kinase	PDK1	Regulation of HIF-1 signaling pathway, inhibition of PDH activity, coordination of glycolysis and TCA cycle, and maintenance of metabolic homeostasis
A0A3Q1MXQ0	0.595685923	0.00518677	Phosphopyruvate hydratase	ENO1	Key glycolytic enzyme (catalyzes 2-phosphoglycerate → phosphoenolpyruvate), involved in the regulation of glycolytic flux, and associated with meat color stability
A1A4J1	0.615333034	0.019027324	ATP-dependent 6-phosphofructokinase, liver type	PFKL	Rate-limiting enzyme of glycolysis (catalyzes fructose-6-phosphate → fructose-1,6-bisphosphate), regulates glycolytic rate, and maintains postmortem muscle pH
A6QLL8	0.414880604	0.029800935	Fructose-bisphosphate aldolase	ALDOA	Key glycolytic enzyme (catalyzes the cleavage of fructose-1,6-bisphosphate), maintains the flux of intermediate glycolytic metabolism, and correlates with oxymyoglobin retention
A7MB35	0.628383244	0.008241125	Pyruvate dehydrogenase E1 component subunit alpha, somatic form, mitochondrial	PDHA1	Key linking enzyme between glycolysis and TCA cycle (catalyzes pyruvate → acetyl-CoA), coordinates mitochondrial oxidation and glycolysis, and maintains metabolic balance
E1BEJ3	0.868915049	0.002689152	Elongation factor G, mitochondrial	GFM1	Mitochondrial translation elongation factor (with GTPase activity), involved in the synthesis of mitochondrial proteins (ETC complex subunits), and maintains mitochondrial function and meat color stability
P10096	0.466134649	0.040696784	Glyceraldehyde-3-phosphate dehydrogenase	GAPDH	Core glycolytic enzyme, links glycolysis and mitochondrial metabolism, participates in oxidative stress response, and associates with antioxidant enzyme activity
Q0VC21	1.857292463	0.008152637	Large ribosomal subunit protein uL15m	MRPL15	Mitochondrial ribosomal large subunit protein, involved in mitochondrial protein translation, synergizes with GFM1 to maintain mitochondrial function, and correlates with beef redness
Q2KID9	1.485589173	0.016583329	Small ribosomal subunit protein uS5m	MRPS5	Mitochondrial ribosomal small subunit protein, regulates mitochondrial translation efficiency, maintains mitochondrial ETC function, and inhibits lipid peroxidation
Q32PI8	1.533641669	0.012126053	Small ribosomal subunit protein mS27	MRPS27	Mitochondrial ribosomal small subunit protein, involved in mitochondrial protein synthesis, maintains mitochondrial homeostasis, and alleviates protein oxidation
Q3T0P6	0.35732558	0.002347449	Phosphoglycerate kinase 1	PGK1	Key glycolytic enzyme (catalyzes 1,3-bisphosphoglycerate → 3-phosphoglycerate), accompanied by ATP generation, and maintains reductive environment and meat color stability
Q3ZBX6	2.005157074	0.009771504	Large ribosomal subunit protein uL3m	MRPL3	Core protein of mitochondrial ribosomal large subunit, maintains ribosomal structural stability, synergizes with GFM1 to regulate mitochondrial translation, and correlates with oxymyoglobin retention
Q5E9B1	0.790096088	0.001329293	L-lactate dehydrogenase B chain	LDHB	Target protein of HIF-1 signaling pathway, catalyzes lactate → pyruvate, promotes myoglobin reduction, and core-regulates meat color stability
Q5E9F2	2.209472123	0.0023072	Heme oxygenase 1	HMOX1	Regulation of HIF-1 signaling pathway, key enzyme for heme degradation, enhances antioxidant capacity, inhibits protein oxidation, and protects myoglobin redox homeostasis

Note: DAPs were defined as proteins with *P* < 0.05 and absolute fold change ≥1.2 (|log₂FC| ≥ 0.26) consistently across 6, 24, and 48 h postmortem. All listed proteins were significantly upregulated in H_2_O_2_-treated beef. Functional descriptions integrate UniProt official annotations and results of pathway enrichment and physicochemical index correlation in this study.

#### 3.6.2 Enrichment analysis of DAPs

GO enrichment was performed to investigate DAPs with specific biological functions. The enrichment score was calculated as -log10 (*P* value) (a smaller *P* value or FDR value corresponds to a higher enrichment score, indicating a greater degree of enrichment), suggesting that the pathway may hold significant biological importance within this dataset. The top 20 enriched functional terms, ranked by ascending *P* value, were selected to generate bar graphs. As shown in Fig. 7A, the significantly enriched Biological Process terms included biological regulation (GO:0065007), regulation of cellular process (GO:0050794), regulation of biological process (GO:0007186), metabolic process (GO:0050789), and regulation of biosynthetic process (GO:0008152). Among these, metabolic process (GO:0050789) contained the highest number of enriched proteins. Within the CC category (Fig. 7B), precatalytic spliceosome (GO:0071011), mitochondrion (GO:0005739), and nucleus (GO:0005634) were the main terms. As for the MF domain (Fig. 7C), peroxisome targeting sequence binding (GO:0000268), transcription regulator activity (GO:0140110), RNA binding (GO:0003723), and catalytic activity (GO:0003824) were the most matched terms, among which catalytic activity (GO:0003824) contained the highest number of enriched proteins.

**Fig. 7:**
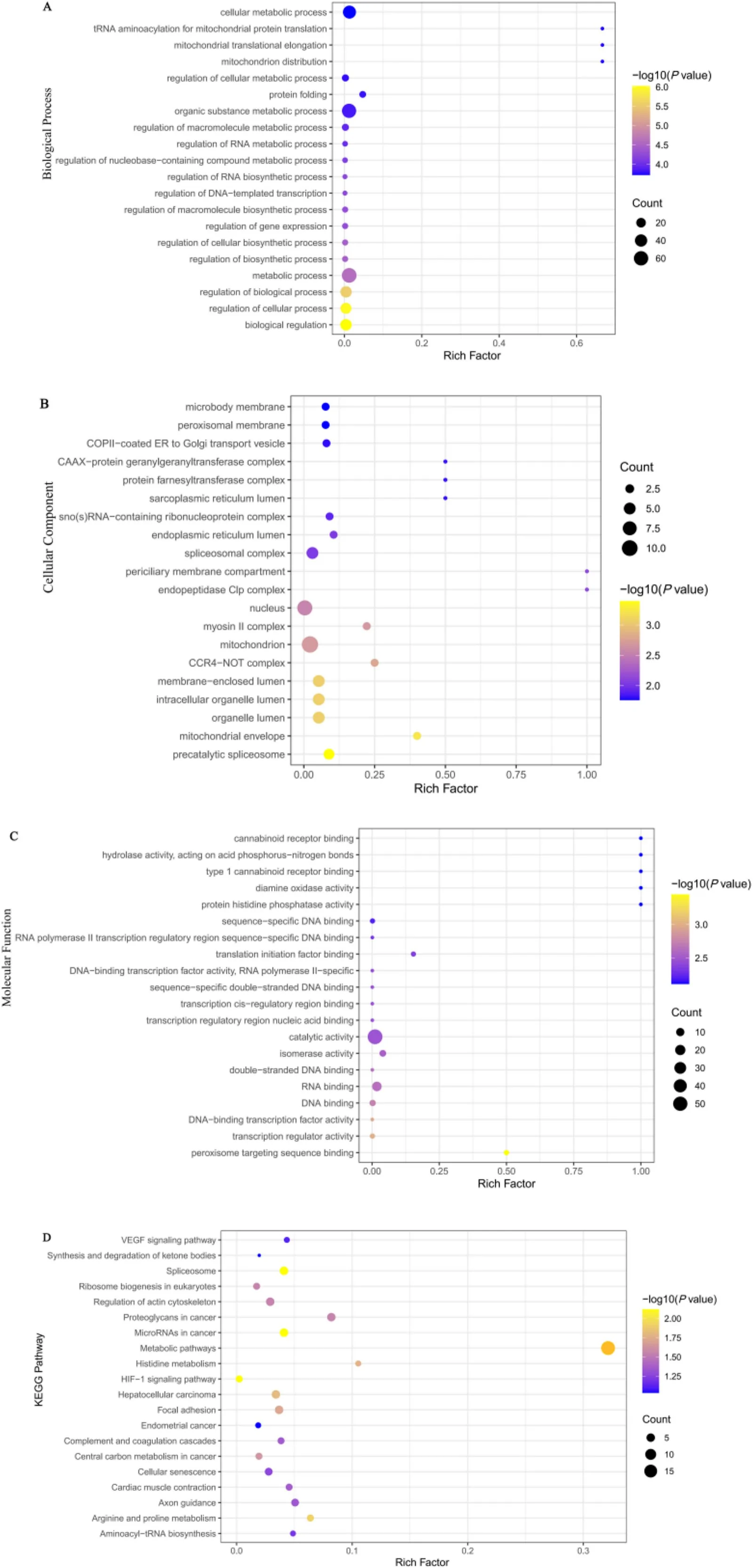
Functional enrichment analysis of the 149 common DAPs identified between H_2_O_2_-treated (500 μmol/L) and control beef across all three postmortem time points (6, 24, and 48 h). A, Biological Process (BP); B, Cellular Component (CC); C, Molecular Function (MF); D, KEGG pathway enrichment. The horizontal axis represents the Rich Factor (the ratio of DAPs annotated to a term/pathway to the total proteins annotated to that term/pathway). The color scale represents -log10 (*P* value), with darker colors indicating more statistically significant enrichment. Only terms with FDR < 0.05 were considered significantly enriched. The size of each bubble represents the number of DAPs enriched in that term/pathway.

To better understand the biological metabolic pathways of the differential proteins and explain the molecular mechanism behind the improved color stability in beef treated with 500 μmol/L H_2_O_2_, this study employed KEGG pathway analysis to investigate the enrichment of major metabolic pathways, with a focus on DAPs. As shown in Fig. 7D, the most significantly enriched pathways compared to the control group were VEGF signaling pathway, MicroRNAs in cancer, Spliceosome, HIF-1 signaling pathway, Arginine and proline metabolism, and Metabolic pathways (*P* < 0.05). Notably, three proteins were enriched in Ribosome biogenesis in eukaryotes, and three proteins (PDK1, HMOX1, and LDHB) were enriched in the HIF-1 signaling pathway (*P* < 0.01). The Metabolic pathways category contained the highest number of differentially abundant proteins. These significantly upregulated pathways and DAPs will be discussed in detail in the following section.

#### 3.6.3 Protein-protein interaction (PPI) of DAPs

Based on the key DAPs listed in Table 2, a protein-protein interaction (PPI) network was constructed to visually represent their potential relationships and functional roles underlying beef color stability under 500 μmol/L H_2_O_2_ treatment. Only experimentally validated interactions with a confidence score > 0.7 were included in the final network to ensure the reliability of the predicted protein-protein associations. All differentially expressed proteins were extracted from the STRING 10.0 database to construct the PPI network, which contained 48 edges and 27 nodes (Fig. 8A). The network revealed two major functional clusters. The first cluster, with LDHB as the hub protein, contained 8 nodes (LDHB, PFKL, GAPDH, ENO1, PDHA1, ALDOA, PGK1 and PDK1) and 24 edges. This cluster was primarily involved in cellular metabolic processes, catalytic activity, and the regulation of the HIF-1 signaling pathway. The second cluster, centered on GFM1, consisted of 9 nodes (GFM1, GAPDH, ENO1, PDHA1, PGK1, MRPL3, MRPS5, MRPL15 and MRPS27) and 19 edges. Its components mainly participated in the primary metabolic process and showed significant upregulation predominantly in the mitochondrion. Within this second cluster, a tight submodule comprising GFM1 and four mitochondrial ribosomal proteins (MRPL3, MRPS5, MRPL15 and MRPS27) was observed, containing 5 nodes and 12 edges. This submodule was primarily associated with the mitochondrial protein-containing complex and mitochondrial ribosome.

**Fig. 8:**
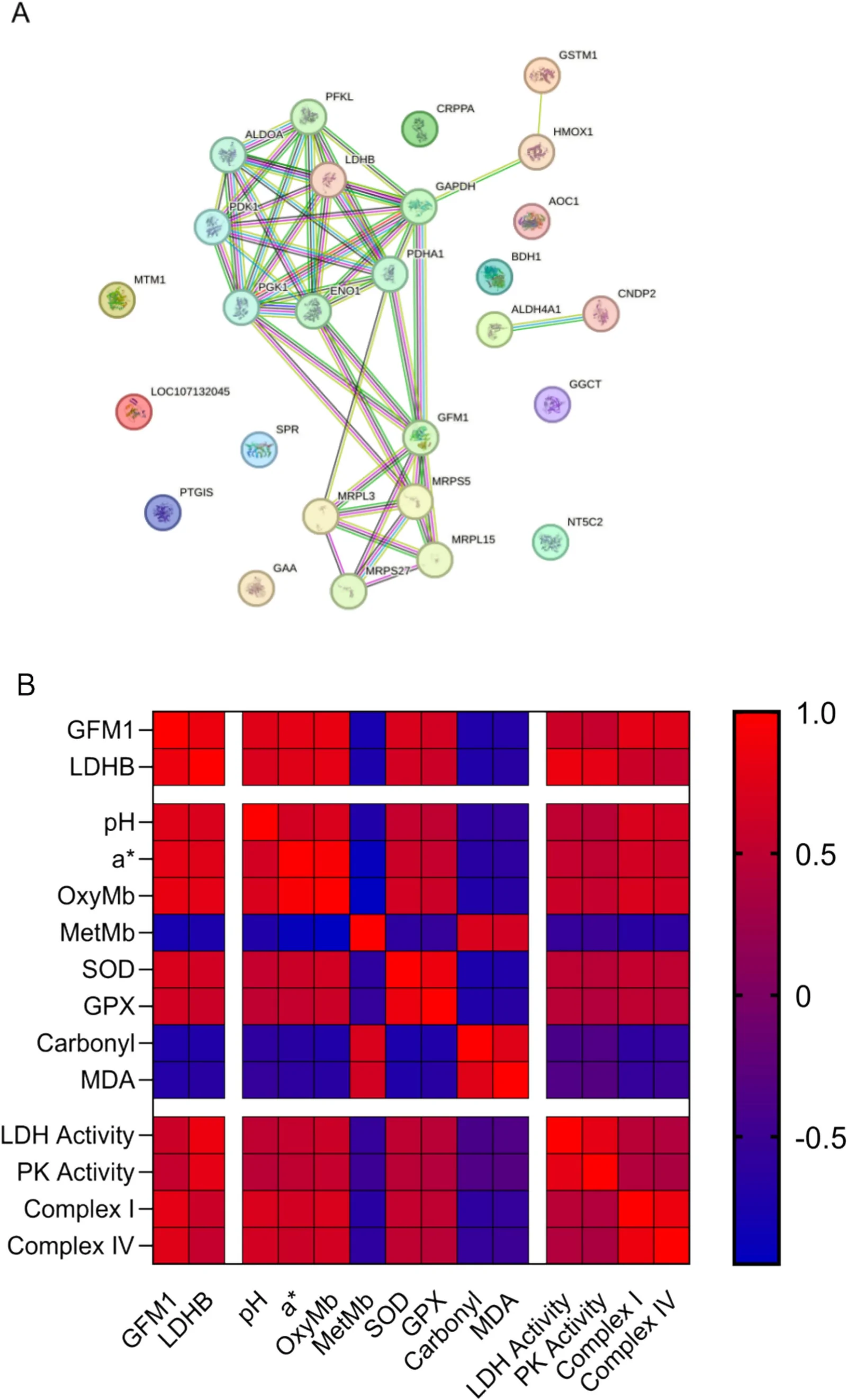
Protein-protein interaction (PPI) network of key DAPs (A) and Pearson correlation analysis between key DAPs and physicochemical indicators (B) in postmortem beef under H_2_O_2_-induced oxidative stress (500 μmol/L, *n* = 3 biological replicates per time point, pooled across 6, 24, and 48 h for correlation analysis, total *n* = 24). A, PPI network constructed using STRING database with only experimentally validated interactions (confidence score > 0.7). Nodes represent proteins; edges represent interactions. B, Correlation heatmap showing Pearson correlation coefficients (r) between 13 key DAPs and physicochemical indicators. The color scale ranges from −1 (blue, strong negative correlation) to +1 (red, strong positive correlation). Numerical values are shown for correlations with |r| > 0.3. Asterisks (*) indicate statistically significant correlations (*P* < 0.05). (For interpretation of the references to color in this figure legend, the reader is referred to the web version of this article.)

### 3.7 Correlation between key DAPs and physicochemical indicators

Pearson correlation analysis was performed between the key DAPs listed in Table 2 and all measured physicochemical indicators to explore their functional associations in postmortem beef under H_2_O_2_-induced oxidative stress (Fig. 8B; Table S2). The resulting heatmap revealed distinct clustering patterns, with proteins segregating into functional modules that corresponded with their biological roles. The mitochondrial-associated proteins, particularly GFM1, demonstrated strong positive correlations with mitochondrial complex activities (Complex I: *r* = 0.83; Complex IV: *r* = 0.80) and color stability parameters including a* value (*r* = 0.84) and oxymyoglobin percentage (*r* = 0.86). Concurrently, this protein cluster showed significant negative correlations with metmyoglobin accumulation (*r* = − 0.85) and oxidative damage markers, including protein carbonyl content (*r* = − 0.78) and MDA levels (*r* = − 0.76). The mitochondrial ribosomal proteins (MRPL3, MRPS5, MRPL15, MRPS27) exhibited similar correlation patterns, though with slightly lower coefficients, suggesting coordinated function within this module. The glycolytic protein cluster, centered around LDHB, displayed strong positive correlations with glycolytic enzyme activities, most notably with LDH activity itself (*r* = 0.89) and pyruvate kinase activity (*r* = 0.84). This module also showed moderate positive correlations with antioxidant enzyme activities (SOD: *r* = 0.68; GPX: *r* = 0.71) and color stability indicators (a* value: *r* = 0.81; OxyMb: *r* = 0.83). Interestingly, both mitochondrial and glycolytic protein modules demonstrated positive correlations with ultimate pH values (*r* = 0.58–0.79), suggesting a coordinated regulation of postmortem acidification. Proteins at the metabolic interface, including GAPDH, ENO1, and PDHA1, exhibited intermediate correlation patterns with both mitochondrial and glycolytic parameters. PDHA1, which links glycolysis to mitochondrial oxidation, showed substantial positive correlations with mitochondrial complex activities (*r* = 0.57–0.72) while maintaining moderate correlations with glycolytic enzymes. The correlation network thus reveals two functionally coordinated protein systems that appear to work in concert to maintain color stability under oxidative stress conditions, with GFM1 and LDHB serving as central nodes in their respective modules.

## 4 Discussion

Meat color is a critical quality attribute determining consumer purchasing decisions, and its stability is intricately regulated by postmortem muscle metabolism and oxidative stress status (Patinho et al., 2024). This study, by applying precisely defined concentrations of exogenous H_2_O_2_ as an experimental model of oxidative stress, demonstrated that moderate oxidative stress (500 μmol/L H_2_O_2_) promotes color stability in postmortem beef and thoroughly investigated its association with mitochondrial function and energy metabolism pathways.

We observed that treatment with 500 μmol/L H_2_O_2_ effectively maintained a higher pH value in postmortem beef (Fig. 1) and significantly increased redness and oxymyoglobin proportion while inhibiting metmyoglobin accumulation (Table 1). The redox state of myoglobin directly determines meat color, and pH acts as a key environmental factor regulating metmyoglobin reduction by affecting endogenous enzyme activities and myoglobin structure (Alvarez-Armenta et al., 2023; Zhong et al., 2025). Notably, beef redness is optimal and most stable when muscle pH is maintained within 5.8 to 6.2 (Zhang et al., 2018). Rapid postmortem pH decline accelerates MetMb formation (Liu et al., 2025). In this study, the attenuated pH decline in the 500 μmol/L H₂O₂ group provided a favorable microenvironment for maintaining myoglobin in its reduced state, indicating that moderate oxidative stress improves color stability indirectly by regulating overall muscle metabolism rather than directly acting on myoglobin. Extensive studies confirm that pH critically affects beef redness and color stability, as slower acidification and higher ultimate pH enhance a* values and oxymyoglobin accumulation while reducing MetMb formation (Yang et al., 2025). The retarded pH drops caused by 500 μmol/L H_2_O_2_ substantially contributed to improved color stability by preserving mitochondrial electron transport chain activity, sustaining a reducing intracellular environment, and inhibiting myoglobin oxidation (Gagaoua et al., 2020; Patinho et al., 2024). This was further supported by the significantly positive correlation between pH and a* value in our correlation analysis (Fig. 8B), demonstrating that moderate slowing of postmortem pH decline is an important mechanism underlying the enhanced color stability induced by appropriate oxidative stress. In contrast, previous studies using high concentrations of H_2_O_2_ (≥10 mM) reported that severe oxidative stress accelerates glycolysis, activates apoptotic pathways, and ultimately enhances meat tenderization rather than color stability (Wang et al., 2018; Yan et al., 2022; Yang et al., 2024). Unlike those findings, our moderate dose (500 μmol/L) attenuated pH decline, induced adaptive antioxidant responses (increased GPX and SOD, Fig. 2a, b), and preserved mitochondrial electron transport chain activity without triggering overt apoptosis. Both our study and the high-concentration studies observed HIF-1 activation and glycolytic enzyme upregulation, indicating this is a common response to H_2_O_2_. However, the downstream consequences diverge. High doses exacerbate acidification and tenderization, whereas the moderate dose in our study promotes mitochondrial maintenance and redox balance, thereby improving color stability. This concentration-dependent dichotomy underscores the hormetic nature of H_2_O_2_ effects on postmortem muscle, where moderate oxidative stress induces adaptive protection while excessive stress becomes detrimental. A related study by Chen et al. (2022) confirmed similar findings but lacked proteomic data. Our work extends this by revealing that moderate H_2_O_2_ co-upregulates mitochondrial proteins (GFM1, MRPL3, MRPS5, MRPL15, MRPS27) alongside glycolytic enzymes (LDHB, PFKL, PGK1)—a coordinated adaptive program that preserves mitochondrial translation and prevents the excessive acidification seen with unopposed glycolysis.

Although the 1000 μmol/L H_2_O_2_ group maintained significantly higher pH values throughout postmortem storage (Fig. 1), its color stability was generally inferior to that of the 500 μmol/L group, as indicated by lower a* values and OxyMb proportion and higher MetMb formation (Table 1). This observation suggests that simply maintaining a higher pH may not be sufficient to protect meat color under conditions of excessive oxidative stress. Indeed, the 1000 μmol/L treatment caused markedly greater protein carbonyl accumulation and free thiol loss compared to the 500 μmol/L group (Fig. 2d, e), which is indicative of severe oxidative damage to myoglobin and the cellular reducing environment (Setyabrata et al., 2023). Protein oxidation has been reported to directly convert ferrous myoglobin to ferric metmyoglobin and can also impair the activity of metmyoglobin-reducing enzymes (Guo et al., 2024). Consequently, the detrimental effects of excessive oxidative stress appear to override any potential benefit of a higher pH in the 1000 μmol/L group. In contrast, the 500 μmol/L H_2_O_2_ treatment helped to achieve a favorable balance between pH retention and oxidative burden, thereby contributing to optimized color stability. The effect of H_2_O_2_ on postmortem pH decline was concentration-dependent (Fig. 1), revealing a non-linear, biphasic response characteristic of hormesis. The 300 μmol/L treatment accelerated acidification (lower pH than control), consistent with previous reports that 200 μmol/L (Chen et al., 2022) and even 20 mmol/L H_2_O_2_ (Zhu et al., 2022) promote glycolysis and pH decline via ROS-induced HIF-1α or CaMKKβ/AMPK pathways. In contrast, the 500 μmol/L treatment attenuated pH decline despite also activating HIF-1 signaling and glycolytic enzymes—a paradox suggesting that moderate oxidative stress triggers a distinct adaptive response that preserves mitochondrial function and redox balance, counteracting excessive acidification. Importantly, the 1000 μmol/L group, despite achieving the highest pH, exhibited inferior color stability. This seeming contradiction is resolved by examining oxidative damage markers: the 1000 μmol/L treatment caused markedly greater protein carbonylation and free thiol loss (Fig. 2d, e), indicating that oxidative damage to redox-sensitive enzymes (e.g., metmyoglobin reductases) exceeded cellular repair capacity. Although higher pH should theoretically favor myoglobin reduction, the severe oxidative modification of the reducing machinery itself rendered the system incapable of utilizing this favorable pH environment. Thus, the protective effect of elevated pH is conditional on redox network integrity—when oxidative damage surpasses a critical threshold, the pH benefit is negated. Overall, 300 μmol/L was insufficient to trigger adequate adaptive responses, 500 μmol/L optimally induced hormetic protection (enhanced GPX/SOD, preserved mitochondrial function, attenuated pH decline), while 1000 μmol/L caused excessive oxidative damage that overrode any pH benefit—a pattern consistent with the hormesis framework (Calabrese, 2016).

The balance between oxidative stress and the antioxidant defense system is central to meat color stability (Li et al., 2025b). Our results showed that the 500 μmol/L H_2_O_2_ treatment specifically enhanced the activities of GPX and SOD (Fig. 2a, b). This likely represents an adaptive response, where the cell, upon sensing a moderate oxidative threat, initiates endogenous antioxidant programs to maintain redox homeostasis (Yang et al., 2024). This activation effectively controlled the level of lipid peroxidation, as evidenced by the significantly lower MDA content in this treatment group compared to the control and 300 μmol/L groups (Fig. 2f). Although signs of protein oxidation (decreased free thiol content, Fig. 2e) were also present in this group, the upregulated antioxidant enzyme system potentially prioritized mitigating lipid oxidation, which contributes more significantly to color deterioration. This seemingly contradictory observation can be explained by an adaptive antioxidant response, in which moderate oxidative stress activates cellular defense mechanisms that preferentially target lipid peroxidation, as reflected by the reduced MDA levels (Fig. 2f). The concomitant decrease in free thiol content likely reflects the consumption of protein thiols as part of the antioxidant defense, for example through direct scavenging of reactive oxygen species or as a sacrificial substrate to preserve more critical lipid components (Jones & Sies, 2015). Such a trade-off, where protein oxidation occurs while lipid peroxidation is controlled, has been suggested as part of the hormetic response to moderate oxidative stress (Calabrese, 2016). In contrast, 300 μmol/L H_2_O_2_ was insufficient in efficacy, while 1000 μmol/L likely exceeded the cellular compensatory capacity, leading to a relative decrease in antioxidant system efficiency, consistent with previous studies on the concentration-dependent effects of oxidants (Cao et al., 2020).

A noteworthy finding meriting further discussion is that 500 μmol/L H_2_O_2_, while enhancing the activities of key glycolytic enzymes (Fig. 3), also maintained or even augmented the activities of mitochondrial electron transport chain (ETC) complexes I − IV (Fig. 4). This apparent paradox reveals a refined metabolic regulation in postmortem muscle. The conventional view holds that mitochondrial function declines rapidly within 24 h postmortem, with energy supply becoming 89% dependent on glycolysis (Wang et al., 2022). However, our study indicates that moderate oxidative stress may delay the collapse of mitochondrial function to some extent. The maintained activities of mitochondrial complexes, particularly Complex II and IV (Fig. 4b, d), suggest retained partial oxidative phosphorylation capacity, which could aid in slow substrate consumption and potentially attenuate lactate accumulation rate, thereby explaining the observed retarded pH decline (Krauskopf et al., 2025; Ramos et al., 2024). Concurrently, the upregulated activities of glycolytic enzymes might represent a compensatory or preparatory mechanism to ensure adequate ATP supply during this transition (Chen et al., 2022). This metabolic state, characterized by coexistent enhanced glycolytic flux and maintained mitochondrial function, could constitute the crucial metabolic basis for the effective delay of color deterioration by the 500 μmol/L H_2_O_2_ treatment.

Moving beyond correlative observations, our proteomic analysis provides mechanistic insights into the metabolic phenomena that enhanced mitochondrial maintenance, functional preservation, and adaptive remodeling as a previously underappreciated mechanism for maintaining ETC function under oxidative stress. DAPs were significantly enriched in glycolysis, oxidative phosphorylation, and amino acid metabolism sub-pathways within the broader metabolic pathways category, and these DAPs were predominantly upregulated (Fig. 7D), suggesting key roles for these processes in the H_2_O_2_ response. PPI network analysis further identified two central protein nodes, the mitochondrial translation elongation factor GFM1 and LDHB (Fig. 8A). GFM1 is a mitochondrial GTPase that catalyzes the ribosomal translocation step during mitochondrial translation elongation (Ahmad et al., 2025), acting as a core factor in mitochondrial protein synthesis. Its upregulation, coupled with the co-expression of mitochondrial ribosomal proteins like MRPL3 and MRPS5 (Bacon et al., 2024), strongly implies that 500 μmol/L H_2_O_2_ treatment promoted mitochondrial protein homeostasis, structural stability, and functional preservation rather than necessarily indicating de novo mitochondrial biogenesis. This directly relates to the enhanced ETC complex activities observed in this study, as core subunits of the ETC are encoded by the mitochondrial genome (Cilleros-Holgado et al., 2025). Specifically, upstream regulation involving GFM1 could ensure effective assembly and function of ETC complexes, thereby maintaining mitochondrial membrane potential and energy production efficiency, and reducing excess ROS generated via electron leak (Ramos et al., 2024), which is corroborated by the lower oxidative damage markers in this treatment group (Fig. 2d, e, f).

On the other hand, LDHB catalyzes the reversible, stereospecific interconversion of (*S*)-lactate and pyruvate, coupled with the interconversion of NADH and NAD^+^. Notably, LDHB preferentially catalyzes the forward reaction (lactate → pyruvate), generating NADH in the process (Liu et al., 2026). The catalytic direction of the lactate dehydrogenase reaction is highly sensitive to pH, as directly demonstrated by Robergs et al. (2026), who showed that lactate production (pyruvate reduction) consumes H^+^, leading to an increase in pH, whereas the reverse reaction (lactate oxidation to pyruvate) releases H^+^, decreasing pH. Therefore, a less acidic environment is required to maintain the forward catalytic activity of LDHB. In the present study, the 500 μmol/L H_2_O_2_ group maintained significantly higher pH values (5.65–5.79) throughout postmortem storage (Fig. 1). This attenuated acidification, particularly the avoidance of pH below 5.6, likely helps preserve the forward catalytic activity of LDHB, thereby sustaining NADH generation for metmyoglobin reduction (Guo et al., 2024). Beyond this immediate redox function, LDHB's impact extends to broader metabolic regulation. LDHB activity also influences lactate accumulation—a metabolite increasingly recognized as a signaling molecule that modulates redox status and protein lactylation in postmortem muscle (Liu et al., 2026). By maintaining LDHB in its forward direction, the 500 μmol/L H_2_O_2_ treatment may promote controlled lactate production that sustains NAD^+^/NADH balance without causing excessive acidification. This contrasts with the 300 μmol/L group, where accelerated glycolysis likely led to rapid lactate accumulation, exacerbating pH decline and creating feedback suppression of mitochondrial function. The balanced metabolic state in the 500 μmol/L group, characterized by moderate lactate flux and preserved mitochondrial ETC activity (Fig. 4), thus represents an optimized configuration for color stability. This interpretation is supported by the upregulation of PDHA1 (Table 2), which links glycolysis to the TCA cycle, indicating that pyruvate is not exclusively shunted to lactate but also directed toward mitochondrial oxidation. Its functional cluster closely interacts with glycolytic enzymes (GAPDH, ENO1, PGK1) and showed strong positive correlation with glycolytic flux (Fig. 8B). Notably, LDHB and several glycolytic enzymes were also annotated to the HIF-1 signaling pathway. Postmortem muscle rapidly transitions from aerobic respiration to anaerobic glycolysis and develops progressive hypoxia, which naturally triggers stabilization and nuclear translocation of HIF-1α, thereby upregulating HIF-1 signaling to enhance glycolytic gene expression (Gao et al., 2025; Xin et al., 2022).

Our results confirm that 500 μmol/L H_2_O_2_ also activates HIF-1 signaling, but through a distinctly different mechanism compared with hypoxia. Hypoxia-induced HIF-1 activation is driven by oxygen deficiency, which directly inhibits prolyl hydroxylases and prevents HIF-1α degradation (Jaskiewicz et al., 2022). This pathway predominantly suppresses mitochondrial electron transport chain function, exaggerates the metabolic shift to anaerobic glycolysis, accelerates lactate accumulation and pH decline, and impairs meat color stability (Xin et al., 2022; Zhu et al., 2024). Recent studies further demonstrate that HIF-1α activation promotes ferroptosis in postmortem muscle via ferritinophagy and lactate production, negatively impacting color and water-holding capacity while contributing to tenderness development (Zhu et al., 2025). In contrast, H_2_O_2_-induced HIF-1 activation is postulated to occur via mild oxidative stress rather than oxygen limitation. Based on previous literature, H_2_O_2_ may inhibit PHD activity through transient Fe^2+^ oxidation to trigger adaptive HIF-1 signaling (Sun et al., 2024), however, direct experimental evidence supporting this mechanism was not provided in the present study. Unlike the hypoxic response, this mode preserves mitochondrial structural integrity and ETC complex activities, maintains coordinated pyruvate flux between glycolysis and mitochondrial oxidation, slows pH decline, and enhances antioxidant capacity and metmyoglobin reduction (Li et al., 2019; Chen et al., 2022; Gao et al., 2025), which is consistent with our current findings on HIF-1-mediated meat color regulation (Table 1) and antioxidant defense system (Fig. 2a, b, c). Therefore, although both stimuli upregulate HIF-1 signaling, hypoxia-driven HIF-1 pathway promotes a “hypoxic metabolic adaptation” that accelerates acidification and color deterioration, whereas H_2_O_2_-driven HIF-1 pathway may mediate an “adaptive redox metabolic program” that coordinates mitochondrial maintenance, balanced glycolytic flux, and redox homeostasis to improve beef color stability. While direct quantification of HIF-1α protein was not performed due to the exploratory nature of our proteomic survey, the significant upregulation of HIF-1α regulated glycolytic enzymes (LDHB, PFKL, PGK1, PDK1) and the enrichment of the HIF-1 signaling pathway in KEGG analysis (Fig. 7D) provide indirect but consistent evidence for HIF-1α activation under moderate oxidative stress, consistent with the well-established role of HIF-1α as a master transcriptional regulator of glycolysis. To verify the precise mechanism of H_2_O_2_-induced HIF-1 activation in postmortem beef, future studies are recommended to directly detect HIF-1α protein expression levels and nuclear translocation via Western blotting and immunofluorescence localization, respectively.

Direct application of H_2_O_2_ may not be directly applicable to industrial meat processing due to safety and regulatory considerations. However, the mechanistic insights from this study suggest alternative hypotheses that could be explored in future research to improve beef color stability. It should be emphasized that these potential strategies are derived from mechanistic inference rather than direct experimental validation in this study, and their practical feasibility requires systematic investigation. These include nutritional interventions targeting the GFM1-mediated mitochondrial pathway, such as dietary polyphenols, selenium, or functional amino acids (e.g., arginine, taurine), which have been reported to enhance mitochondrial function and redox capacity (Chen et al., 2022; Tuell et al., 2021). Another approach involves modulating LDHB activity and glycolytic flux via lactate or its precursors to sustain NADH generation for metmyoglobin reduction (Guo et al., 2024). In addition, safe natural pro-oxidants or controlled oxygen packaging could be investigated to induce mild oxidative stress mimicking the hormetic effects of 500 μmol/L H_2_O_2_, thereby activating adaptive antioxidant responses and HIF-1 signaling without the safety concerns of direct H_2_O_2_ application. These potential strategies warrant further validation in future studies under commercial conditions.

Integrating these findings, we propose a mechanistic model for how moderate oxidative stress (500 μmol/L H_2_O_2_) enhances beef color stability. The initial mild oxidative challenge activates a dual adaptive response. First, it stimulates HIF-1 signaling, upregulating glycolytic enzymes (LDHB, PFKL, PGK1) to ensure ATP production during the postmortem energy crisis. Crucially, unlike hypoxia-driven HIF-1 activation, this oxidative stress-induced response is coupled with upregulation of mitochondrial translation factors (GFM1) and ribosomal proteins (MRPL3, MRPS5, MRPL15, MRPS27), which preserve ETC complex activities and prevent excessive acidification. Second, this mild challenge activates antioxidant enzymes (GPX and SOD), limiting lipid peroxidation while allowing controlled protein oxidation that serves as redox signaling. The maintained mitochondrial function, combined with enhanced antioxidant capacity, sustains a reducing intracellular environment, which—together with the less acidic pH—favors LDHB forward activity and continuous NADH production for metmyoglobin reduction. The net effect is increased oxymyoglobin proportion and redness. Lower doses (300 μmol/L) fail to trigger sufficient adaptive responses, while higher doses (1000 μmol/L) overwhelm the system, causing irreversible oxidative damage that negates any pH benefit. This model provides a mechanistic framework for understanding the hormetic regulation of meat color stability and identifies GFM1 and LDHB as key nodes for potential intervention.

## 5 Conclusions

In the present study, an integrated proteomic and biochemical approach elucidated the molecular mechanisms by which H_2_O_2_-induced oxidative stress influences color stability in postmortem beef. The 500 μmol/L H₂O₂ treatment was identified as optimal, significantly enhancing redness (a* value) and oxymyoglobin proportion while suppressing metmyoglobin formation. Mechanistically, this improvement was associated with attenuated pH decline, enhanced activities of antioxidant enzymes (GPX and SOD), and maintained mitochondrial electron transport chain complex (I − IV) activities. Proteomic analysis identified 149 common DAPs, revealing the mitochondrial translation factor GFM1 and lactate dehydrogenase B (LDHB) as central nodes within a coordinated protein network. GFM1 was strongly correlated with mitochondrial complex activities and color stability, supporting its role in maintaining mitochondrial translational capacity and functional integrity under oxidative stress, while LDHB was linked to glycolytic flux and the HIF-1 signaling pathway. Proteins such as PDHA1, GAPDH, and ENO1 functionally connected these mitochondrial maintenance and glycolytic modules, forming an integrated metabolic network. These DAPs represent potential mechanistic biomarkers for beef color stability. Collectively, this study provides a proteomic basis for understanding the adaptive metabolic coordination underlying color regulation. While our findings demonstrate that moderate oxidative stress can serve as a mechanistic tool to probe and potentially enhance fresh beef quality, the current work was conducted under controlled laboratory conditions using exogenous H_2_O_2_ injection. Further studies are needed to validate these mechanisms under commercial processing conditions and to assess the safety, scalability, and long-term stability of any potential applications.

## CRediT authorship contribution statement

**Cai-Yun Li:** Writing – review & editing, Writing – original draft, Visualization, Validation, Supervision, Software, Methodology, Investigation, Formal analysis, Data curation, Conceptualization. **Xiao-Qian Duan:** Visualization, Software, Data curation. **Xin Yu:** Software, Methodology, Investigation, Conceptualization. **Yu-Bin Zhang:** Writing – review & editing, Project administration, Investigation, Funding acquisition, Data curation, Conceptualization.

## Ethics approval

All procedures involving animals were approved by the Institutional Animal Care and Use Committee of Gansu Agricultural University (Project No. 2023-2-159).

## Declaration of competing interest

The authors declare that they have no known competing financial interests or personal relationships that could have appeared to influence the work reported in this paper.

## Data Availability

Data will be made available on request.
